# The Gut Microbiota Regulates Motor Deficits via Butyrate in a *Gnal* ^+/−^ Mouse Model of DYT25 Dystonia

**DOI:** 10.1002/advs.202512942

**Published:** 2025-12-12

**Authors:** Jingya Guo, Seong‐Gook Kang, Kunlun Huang, Tao Tong

**Affiliations:** ^1^ College of Food Science and Nutritional Engineering China Agricultural University Beijing 100083 China; ^2^ Department of Food Engineering and Solar Salt Research Center Mokpo National University Muangun 58554 Republic of Korea; ^3^ Key Laboratory of Safety Assessment of Genetically Modified Organism (Food Safety) The Ministry of Agriculture and Rural Affairs of the PR China Beijing 100083 China

**Keywords:** butyric acid, dystonia, gut metabolites, gut microbiota, movement disorder

## Abstract

Dystonia is the third most common movement disorder, following essential tremor and Parkinson's disease. The underlying mechanisms behind dystonia are still one of the crucial unsolved research topics. *Gnal* haploinsufficient (*Gnal* ^+/−^) mice are used as a model of DTY25 dystonia to investigate the mechanistic relationship between gut microbiota and dystonia. The present study unveiled *Gnal* ^+/−^ mice exhibit significant motor deficits of dystonia, along with a marked gut microbiota dysbiosis. Analysis of the gut microbiota composition and function reveals that *Gnal* ^+/−^ mice have decreased butyrate‐producing bacteria abundance (such as *Lachnospiraceae_NK4A136*, *Blautia*, and *Butyricicoccus*) and disrupted butanoate metabolism. The targeted metabolomics analysis indicates that the *Gnal* ^+/−^ mice exhibit decreased butyrate levels in feces and serum. The colonization of antibiotic‐treated wild‐type mice with fecal microbiota from *Gnal* ^+/−^ mice is sufficient to induce motor deficit symptoms. Oral administration of sodium butyrate ameliorated motor deficits in the *Gnal* ^+/−^ mouse model of DYT25 dystonia. Striatal single‐nucleus RNA sequencing reveals cell‐type‐specific gene expression changes, suggesting that butyrate modulates neurotransmitter pathways, particularly GABA signaling. This is confirmed by restored striatal GABA levels after butyrate supplementation. In sum, gut microbiome contributes to dystonia pathogenesis, and butyrate supplementation alleviates the motor deficits of dystonia in *Gnal* ^+/−^ mice.

## Graphical abstract

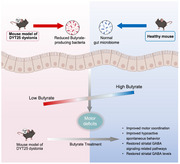

## 1 Introduction

Dystonia is the third most common movement disorder, following essential tremor and Parkinson's disease (PD).^[^ ^1^, ^2^ ^]^ Several varieties of dystonia can lead to severe impairment and a decreased standard of living.^[^ ^3^ ^]^ The underlying mechanisms behind dystonia are still one of the crucial unsolved research topics, as evidenced by the absence of appropriate treatment. Therefore, it is urgent to gain new insights into the pathophysiology of dystonia.

Increasing evidence supports the notion that the gut microbiome has been implicated in the pathophysiology of neurological movement disorders encompassing PD and Huntington's disease (HD).^[^ ^4^, ^5^ ^]^ Within the realm of dystonia, recent studies have reported gut microbial dysbiosis in both clinical cohorts and animal models.^[^ ^6^, ^7^, ^8^ ^]^ A case report of a young woman with myoclonic dystonia showed a remarkable 90% improvement following antibiotic treatment, further hinting at a possible connection between gut microbiota and dystonia symptoms.^[^ ^9^ ^]^ Nevertheless, despite the established link between gut‐brain crosstalk and movement disorders such as PD and HD, the precise contribution of the gut microbiome in the etiology and development of dystonia remains poorly understood.

Here, the aim of the present study is to reveal the underlying mechanistic linkage between gut microbiota and dystonia pathophysiology, as well as to explore prospective therapeutic strategies. To this end, we utilized a *Gnal* ^+/−^ mouse model of dystonia type 25 (DYT25). The *Gnal* ^+/−^ mouse expresses ≈50% of the protein at normal levels and is a good model for mimicking the genetic status of patients with DYT25 dystonia.^[^ ^10^, ^11^ ^]^ Dominant mutations of GNAL genes have been recently identified to be a cause of DYT25 dystonia, and were the first gene linked to adult‐onset focal/segmental inherited dystonia.^[^ ^11^, ^12^ ^]^ Using the *Gnal* ^+/−^ mouse model, we demonstrated the pivotal role of gut‐brain crosstalk in dystonia. *Gnal* ^+/−^ mice exhibited obvious motor deficits of dystonia, along with a maladjusted gut microbial profile. Colonization with fecal microbiota from *Gnal* ^+/−^ mice was sufficient to induce motor deficits in wild‐type (WT) mice. Compared to WT littermates, metabolomic analysis of both serum and feces of *Gnal* ^+/−^ mice showed a significant reduction in butyrate levels. Of therapeutic relevance, oral supplementation with sodium butyrate in *Gnal* ^+/−^ mice effectively rescues the motor deficits phenotypes of DYT25 dystonia. Single‐nucleus RNA sequencing and metabolomics analysis revealed that the butyrate‐treated *Gnal* ^+/−^ mice displayed an upregulated GABA signaling pathway and an elevation of GABA levels in the striatum. These discoveries underscore the role of gut dysbiosis in motor deficits of dystonia pathophysiology and suggest a potential intervention approach to dystonia.

## 2 Results

### 2.1 Gnal+/− Mice Exhibit Motor Deficits of Dystonia

**Figure 1:**
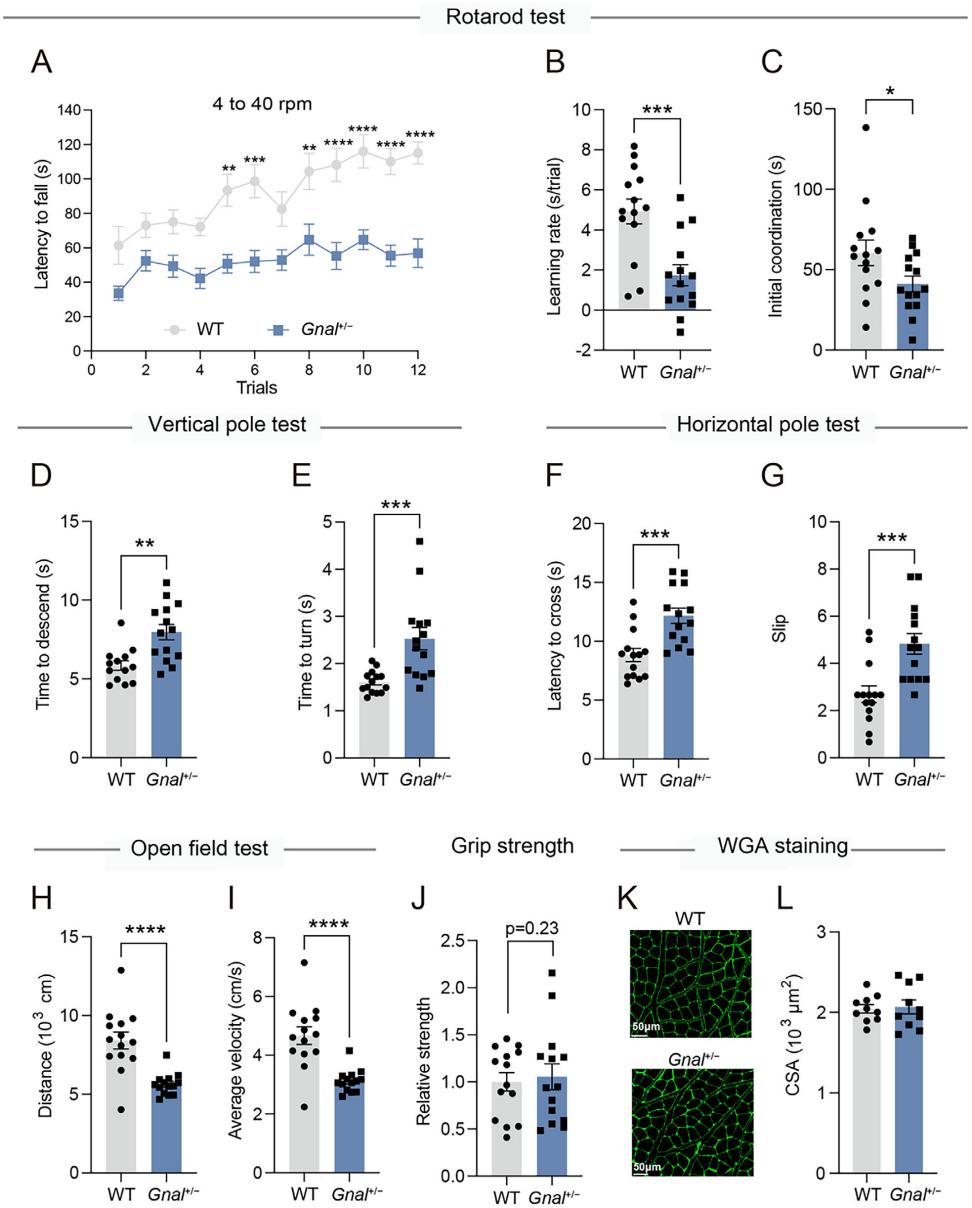
Gnal+/− mice exhibit motor deficits of dystonia. A) Rotarod performance (latency to fall) across 12 testing sessions of WT and *Gnal* ^+/−^ mice littermates. B) Learning rate was derived from the slope value of linear regression performed on data from each individual mouse. C) Initial coordination derived from the intercept value of linear regression performed on data from each individual mouse. D,E) Time to descend (D) and time to turn (E) in the vertical pole test of WT and *Gnal* ^+/−^ mice littermates. F,G) Latency to cross (F) and slip G) in the horizontal pole test of WT and *Gnal* ^+/−^ mice littermates. H,I) Spontaneous locomotor behavior of WT and *Gnal* ^+/−^ mice littermates as assessed by the total distance (H) and average velocity (I). J) Grip strength of the four limbs of WT and *Gnal* ^+/−^ mice littermates. K,L) Representative WGA staining sections of gastrocnemius muscle from WT and *Gnal* ^+/−^ mice (K) (green, scale bars = 50 µm) and CSA of myofibers (L) (n = 10 mean areas from 5 mice per group). All mice were 12–15 months old. Data are shown as means ± SEM (n = 14 per group for A‐J). ^*^ *p* < 0.05, ^**^ *p* < 0.01, ^***^ *p* < 0.001, ^****^ *p* < 0.0001. WGA, wheat germ agglutinin; CSA, cross‐section area.

In the animal model of dystonia, motor coordination is one of the most affected functions.^[^ ^13^, ^14^ ^]^ To systematically evaluate the motor coordination performance of *Gnal* ^+/−^ mice, we performed a battery of behavioral tests. In the acceleration rotarod test, *Gnal* ^+/−^ mice and their corresponding age‐mated WT littermates were subjected to “standard” conditions, with the rotation speed accelerated over 300 s from 4 to 40 rpm.^[^ ^15^ ^]^ Compared to WT littermates, *Gnal* ^+/−^ mice exhibited a decreased latency to fall off the accelerating rotarod in the overall trials (**Figure** **1**A). We subsequently performed a linear regression analysis on data from each mouse, calculating the intercept to estimate initial motor coordination and the slope to assess the learning rate. The *Gnal* ^+/−^ mice displayed significantly decreased initial coordination and learning rates compared to WT mice (Figure 1B,C), which is consistent with previous reports indicating that the *Gnal* ^+/−^ mice display motor coordination impairments.^[^ ^11^ ^]^We further assessed motor coordination performance using two pole tests. In the vertical pole test, *Gnal* ^+/−^ mice took a longer time to descend and turn compared to WT mice (Figure 1D,E). Consistently, in the horizontal pole test, *Gnal* ^+/−^ mice exhibited a significant increase in the latency to cross and the number of slips (Figure 1F,G). Next, the *Gnal* ^+/−^ mice exhibited hypoactive spontaneous behavior, as indicated by a reduction in total distance traveled and average velocity (Figure 1H,I). In the grip strength tests, no significant difference in muscle strength performance was observed between *Gnal* ^+/−^ and WT mice (Figure 1J). We also found no difference in the myofiber area of skeletal muscle between *Gnal* ^+/−^ and WT mice by WGA staining. (Figure 1K,L). These results suggested that motor deficits of *Gnal* ^+/−^ mice may not be caused by decreased muscle strength. In addition, no significant differences were observed in body weight (Figure S1A, Supporting Information) or serum chemistry parameter levels including AST, ALT, GLU, LDL, TC, TG (Figure S1B–G, Supporting Information), between *Gnal* ^+/−^ and WT mice. Taken together, these findings indicate that *Gnal* ^+/−^ mice display motor deficits of DYT25 dystonia, urging deeper exploration into the molecular and neural circuit alterations driving these deficits.

### 2.2 Gnal+/− Mice Manifest a Maladjusted Gut Microbial Profile

**Figure 2:**
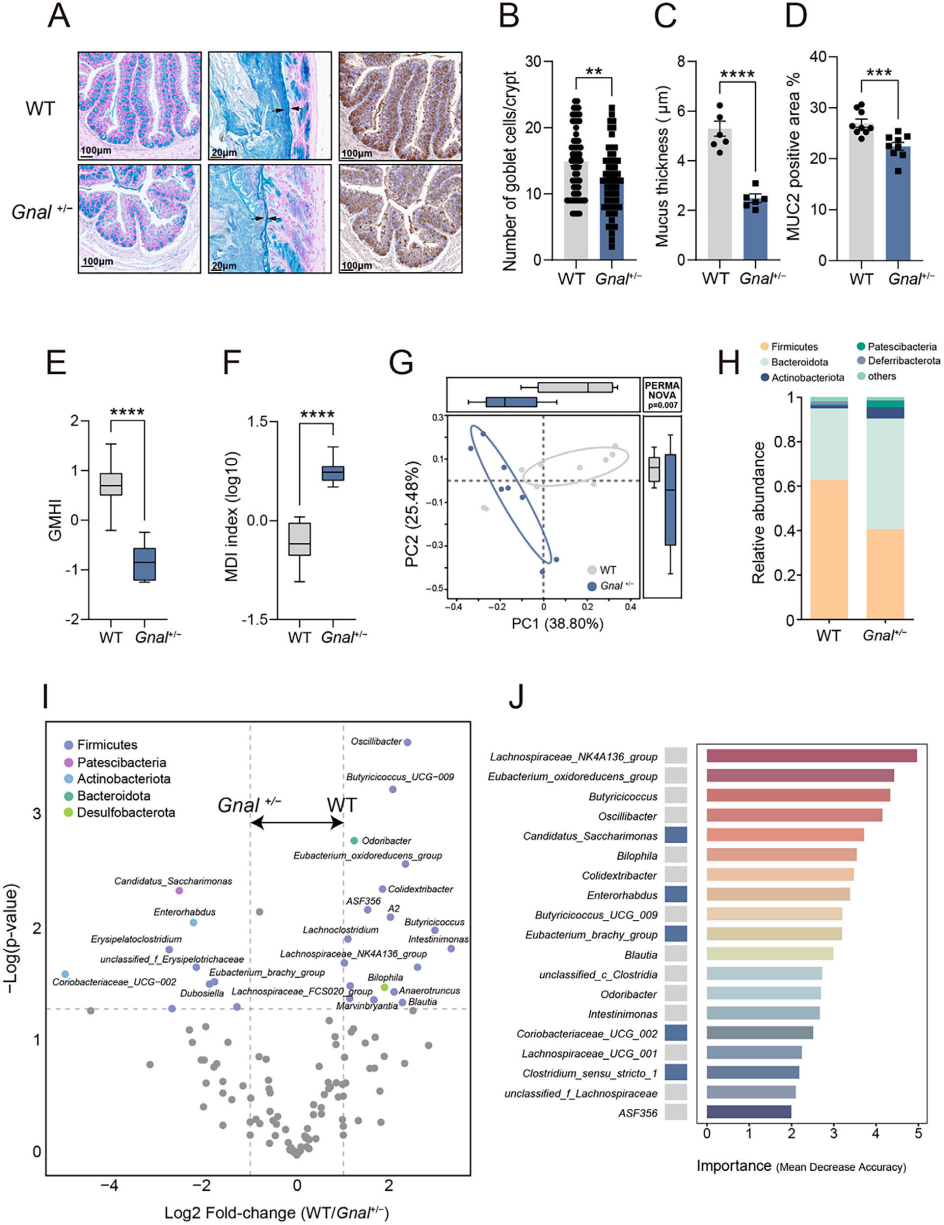
Gnal+/− mice manifest a maladjusted gut microbial profile. A) Representative alcian blue‐stained sections of colon (first and second panel from the left) and representative immunohistochemistry staining of the goblet cells marker MUC2 in colonic sections (third panel from the left). B) Quantification of colonic goblet cell mucus by alcian blue staining per crypt in mice from (A) (first panel from the left) (n = 20 crypts/mouse and 3 mouse group^−1^). C) Quantification of mucus thickness of colonic tissue from mice (n = 6 slices from 3 mice). D) Quantification of the positive area of MUC2 in mice (n = 9 slices from 3 mice). E,F) Gut microbiome indexes of GMHI (E) and MDI (F) in WT and *Gnal* ^+/−^ mice (n = 8 per group). G) β‐diversity of the gut microbiome of WT and *Gnal* ^+/−^ mice as determined by PCoA of BrayCurtis distances (PERMANOVA: *p* = 0.007) (n = 8 per group). H) Averaged relative abundance of bacteria at the phylum level. I) Volcano plot of differential bacterial abundance analysis calculated by Welch's *t*‐test from 16S rRNA gene sequencing. Fold change as a factor, FDR corrected *P* values were plotted for each taxon. Significantly different taxa (FC > 1, *p* < 0.05) were colored according to their phylum. J) Microbiome genus features contributing >2% to classification between WT and *Gnal* ^+/−^ mice by RandomForest analysis. Left: Rectangles indicate taxa with high abundance in WT (shown in gray) or *Gnal* ^+/−^ (shown in blue). Right: Taxon were ordered by their contribution to the correct classification of microbiomes. Data are shown as means ± SEM. ^**^ *p* < 0.01, ^***^ *p* < 0.001, ^****^ *p* < 0.0001. GMHI, gut microbiome health index; MDI, microbial dysbiosis index; PCoA, principal coordinate analysis; PERMANOVA, permutational multivariate analysis of variance.

Recent studies suggest a link between gut microbiota and neurodevelopmental diseases, including PD, AD, and autism spectrum disorder.^[^ ^16^ ^]^ To understand how *Gnal* haploinsufficiency induced behavioral changes, we looked for primary clues by examining gut local changes. We first evaluated the histological alterations in the colon of *Gnal* ^+/−^ mice. As assessed by HE and IHC stainings, we found no significant differences in overt morphology changes and tight junction protein expressions (ZO‐1, Claudin‐1, and Occludin) between *Gnal* ^+/−^ and WT mice (Figure S2A–D, Supporting Information). Notably, the Alcian blue staining and MUC2 immunohistochemical staining revealed that *Gnal* ^+/−^ mice exhibited a significant decrease in goblet cell density, mucus thickness, and MUC2 immunostaining in the colon, compared to WT mice (**Figure** **2**A–D). We further conducted 16S rRNA sequencing on the gut microbiome. While there were no significant changes in α‐diversity (Figure S2E–I, Supporting Information), we calculated the recently developed gut microbiome health index (GMHI) and microbial dysbiosis index (MDI) and found significant differences in GMHI and MDI between *Gnal* ^+/−^ and WT (Figure 2E,F). Compared to traditional α‐diversity, these indexes can distinguish healthy from nonhealthy individuals more reliably.^[^ ^17^, ^18^ ^]^ β diversity analysis revealed clear clustering of samples by genotype, and PERMANOVA analysis demonstrated a significant difference between *Gnal* ^+/−^ and WT groups (Figure 2G).

At the phylum level, Bacteroidota and Firmicutes were mainly abundant in *Gnal* ^+/−^ mice (Figure 2H). At the genus level, differential bacterial taxa between WT and *Gnal* ^+/−^ microbiota were identified and visualized with a Volcano map. Those taxa belong predominantly to the Firmicutes phylum, with single representatives from the Patescibacteria, Actinobacteriota, Bacteroidota, and Desulfobacterota phyla. Specifically, *Candidatus_Saccharimonas*, Eubacterium_brachy_group, and *Coriobacteriaceae_UCG‐002* were prevalent in *Gnal* ^+/−^ group (Figure 2I). Conversely, genera such as *Oscillibacter, Butyricicoccus _UCG_009, Butyricicoccus*, and *Odoribacter* were prevalent in WT mice (Figure 2I). These findings were further validated through an unsupervised Random Forest classification analysis (Figure 2J). Of note, the gut microbiota of *Gnal* ^+/−^ mice exhibited a significant reduction in butyrate‐producing genera, such as *Lachnospiraceae_NK4A136*, *Blautia*, and *Butyricicoccus* (Figure 2I,J). These results demonstrated that *Gnal* ^+/−^ mice exhibited gut microbiota dysbiosis, as evidenced by changes in β diversity and composition of gut microbiota, specifically a notable decrease in butyrate‐producing genera.

**Figure 3:**
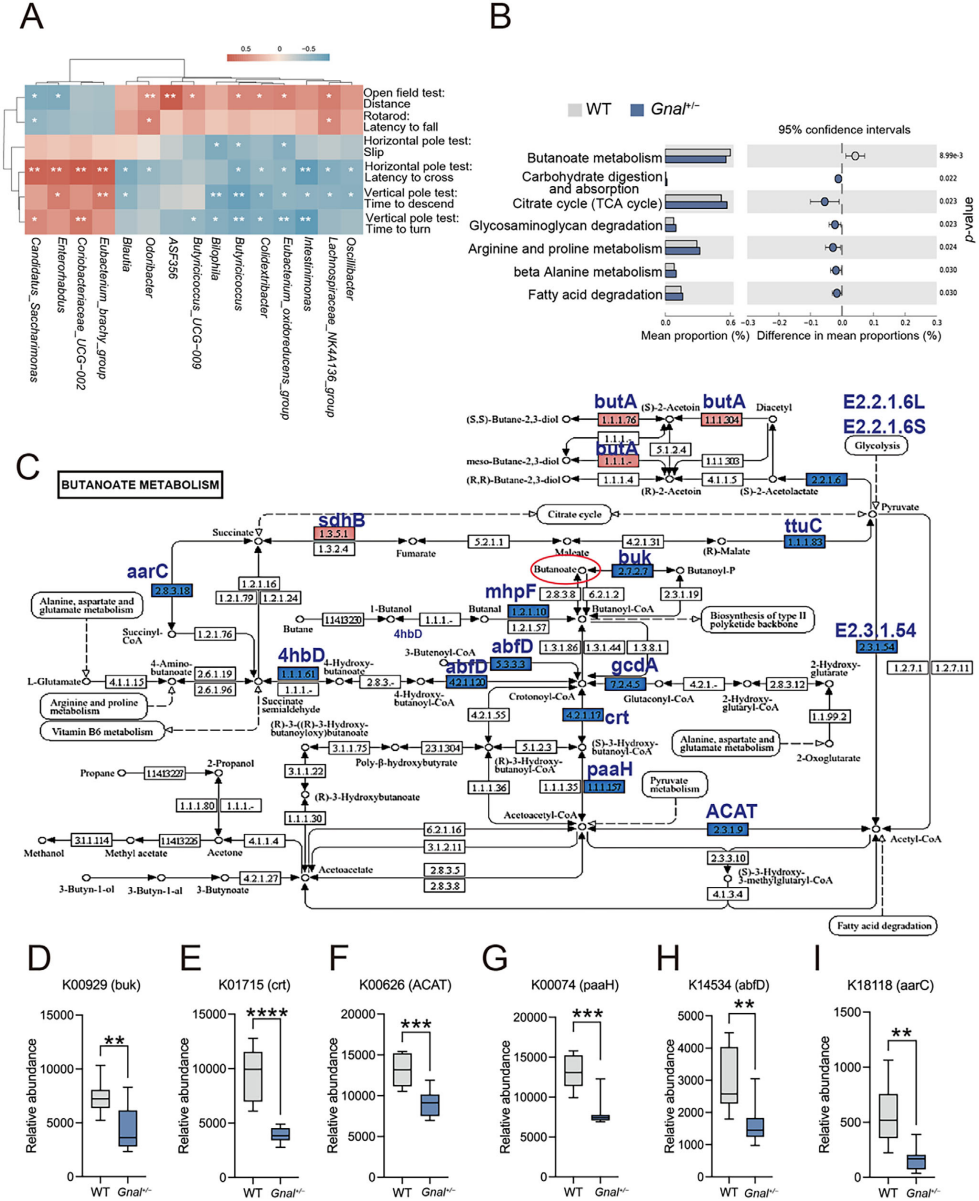
Alterations of gut microbial functions in Gnal+/− mice. A) The abundance of differential taxa in the microbiome was correlated with the behavior of mice. Spearman's r rank correlation between the microbiome and mouse behavior, *P* value < 0.05 for significant correlations was noted. The color scale denotes Spearman's r from red (positive correlation) to blue (negative correlation). B) Differentially enriched functions in the WT (grey) and *Gnal* ^+/−^ (blue) mice with (left) mean proportions of functional items and (right) 95% confidence intervals in the enriched group. C) The metabolic pathway for butyrate metabolism (KEGG pathway map00650) and corresponding KOs were displayed, with a significant decrease in the *Gnal* ^+/−^ group shown in blue and an increase in red. D–I) Box plots of the relative abundance of differential KOs in the butyrate metabolism. Data are shown as means ± SEM. ^**^ *p* < 0.01, ^***^ *p* < 0.001, ^****^ *p* < 0.0001. KEGG, Kyoto Encyclopedia of Genes and Genomes; KO, KEGG orthology.

Next, we investigated whether differential taxa positively or negatively co‐vary with behavioral outcomes, using Spearman's rank correlation. The prevalent taxa in *Gnal* ^+/−^ group correlated with reduced spontaneous locomotor activity and motor coordination deficits behavior. Conversely, the prevalent taxa in the WT group, including *Butyricicoccus*, *Eubacterium_oxidoreducens_group*, *Intestinimonas*, and *Lachnospiraceae_NK4A136_group* showed the opposite effects, as it correlated with increased spontaneous locomotor activity and motor coordination behavior (**Figure** **3**A). To explore how changes in the gut microbiota affected the metabolism of intestinal contents, we performed a microbiota functional analysis. Then, we found that butanoate metabolism (KEGG map00650) differentiated between *Gnal* ^+/−^ mice and WT (Figure 3B,C). A series of KOs were found to be significantly enriched in the butanoate metabolism pathway (Figure 3D–I and Figure S2J–R, Supporting Information). Compared with that of the WT, the gut microbiome of *Gnal* ^+/−^ mice may exhibit decreased potential to produce butyrate, as evidenced by the significantly decreased relative abundance of key butyric acid synthesis genes, such as K00929 (butyrate kinase, buk) and K01715 (enoyl‐coa hydratase, crt) (Figure 3D,E). Overall, these observations suggest that *Gnal* ^+/−^ mice and WT have discrete gut microbial profiles.

### 2.3 Gut Microbiota from Gnal+/− Mice is Sufficient to Promote Motor Deficits of Dystonia

**Figure 4:**
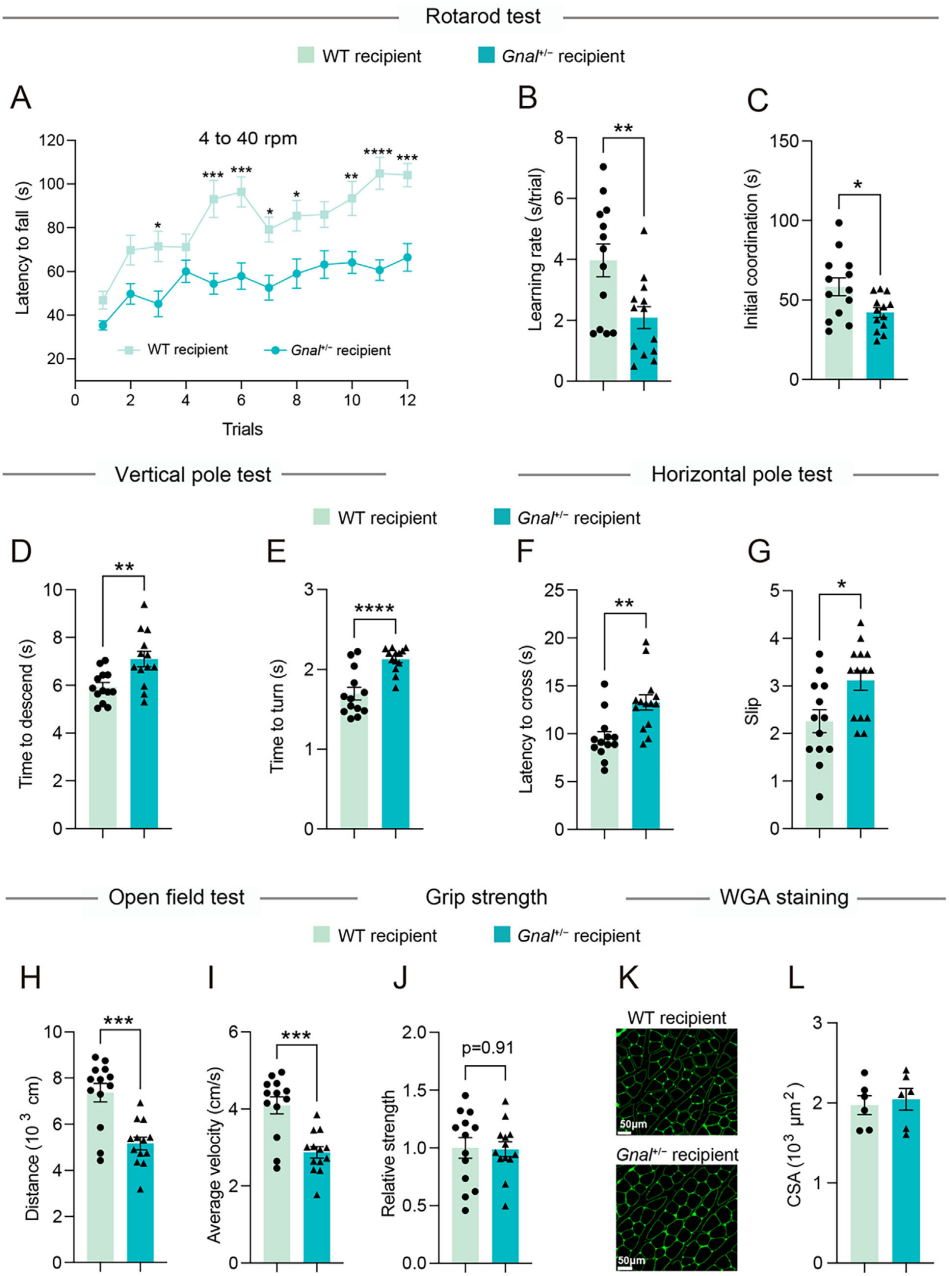
Gut microbiota from Gnal+/− mice is sufficient to promote motor deficits of dystonia. A) Rotarod performance (latency to fall) across 12 testing sessions of male WT recipient and *Gnal* ^+/−^ recipient mice. B) The learning rate was derived from the slope value of linear regression performed on data from each individual mouse. C) Initial coordination derived from the intercept value of linear regression performed on data from each individual mouse. D,E) Time to descend (D) and time to turn (E) in the vertical pole test of WT recipient and *Gnal* ^+/−^ recipient mice. F,G) Latency to cross (F) and slip (G) in the horizontal pole test of WT recipient and *Gnal* ^+/−^ recipient mice. H,I) Spontaneous locomotor behavior of WT recipient and *Gnal* ^+/−^ recipient mice as assessed by the total distance (H), and average velocity (I). J) Grip strength of the four limbs of WT recipient and *Gnal* ^+/−^ recipient mice. K,L) Representative WGA staining sections of gastrocnemius muscle from WT recipient and *Gnal* ^+/−^ recipient mice (K) (green, bars = 50 µm) and CSA of myofibers (L) (n = 6 mean areas from 3 mice per group). All mice were 12–15 months of age. Data are shown as means ± SEM (n = 13 per group for A‐J). ^*^ *p* < 0.05, ^**^ *p* < 0.01, ^***^ *p* < 0.001, ^****^ *p* < 0.0001. WGA, wheat germ agglutinin; CSA, cross‐section area.

To further understand the relationship between gut microbial alternations and dystonia behaviors, we performed fecal microbiota transplantation (FMT) from WT and *Gnal* ^+/−^ donors into antibiotics (ABX)‐treated recipients (labeled “WT recipient” or “*Gnal* ^+/−^ recipient”, respectively). In a series of behavioral tests, we found that the behavior of the recipient mice largely mirrored that of their respective microbiota donors. Specifically, in the rotarod test, *Gnal* ^+/−^ recipient mice exhibited motor coordination deficits, as evidenced by a decreased latency to fall off the rotarod compared with WT recipients (**Figure** **4**A). By calculating the intercept and the slope of the linear regression curve, we found that *Gnal* ^+/−^ recipient mice displayed a significantly decreased initial coordination, with a reduction in learning rate (Figure 4B,C). In the two‐pole tests, compared to WT recipient mice, the *Gnal* ^+/−^ recipient mice had a significant increase in time to descend and turn in the vertical pole test (Figure 4D,E), as well as a significant increase in latency to cross and slips in the horizontal pole test (Figure 4F,G). In the open field test, *Gnal* ^+/−^ recipient mice displayed hypoactive behavior, as indicated by a reduction in total distance traveled and average velocity (Figure 4H,I). Consistent with the *Gnal* ^+/−^ mice, there were no significant differences in muscle strength performance (Figure 4J) and myofiber area (Figure 4K,L) between *Gnal* ^+/−^ recipient and WT recipient mice. Together, these findings support that dysbiosis of the gut microbiome contributes to pathologies in DYT25 dystonia.

### 2.4 Gnal+/− and WT Mice Possess Distinct SCFA Metabolites that Correlate with Behaviors

**Figure 5:**
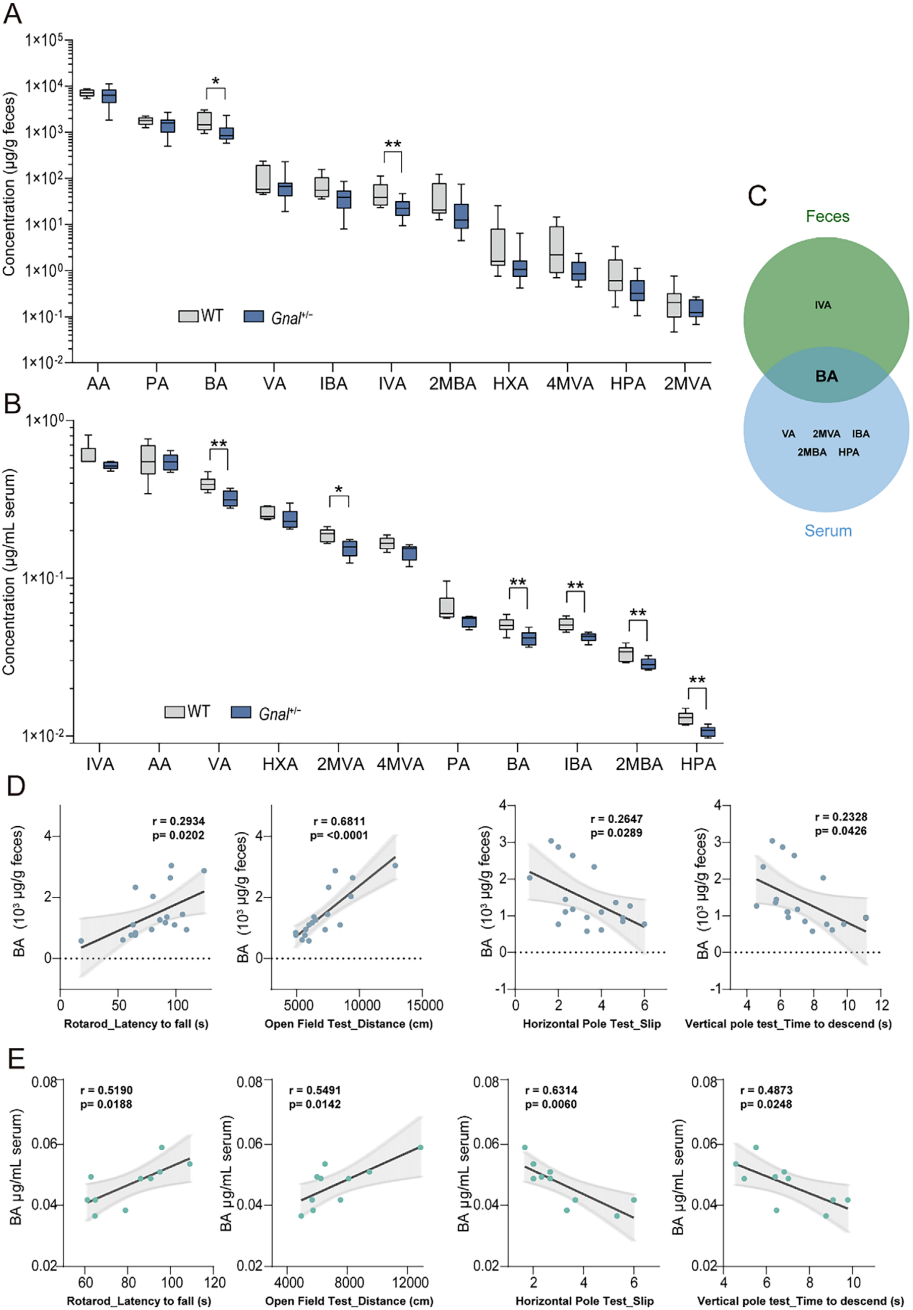
Gnal± mice exhibit profound alterations in gut microbial metabolites. A,B) The concentrations of SCFAs in feces (A) and serum (B) samples of WT and *Gnal* ^+/−^ mice were measured by GC‐MS (n = 9 for feces per group, n = 5 for sera per group). C) The Venn diagram of the significantly differential metabolites in feces and serum. D) Spearman correlations (*p* < 0.05) between BA levels from feces and behavioral parameters. E) Spearman correlations (*p* < 0.05) between BA levels from serum and behavioral parameters. Data are shown as means ± SEM. ^*^ *p* < 0.05, ^**^ *p* < 0.01. AA, acetic acid; PA, propionic acid; BA, butyric acid; VA, valeric acid; IBA: isobutyric acid; IVA: isovaleric acid; 2MBA, 2‐methylbutyric acid; HXA, hexanoic acid; 4MVA, 4‐methylvaleric acid; HPA, heptanoic acid; 2MVA, 2‐methylvaleric acid.

Emerging evidence underscores the importance of gut microbiota‐associated metabolites in influencing host behavior.^[^ ^19^ ^]^ Microbiota functional analysis provides clues that the butanoate metabolism pathway was differentiated between *Gnal* ^+/−^ mice and WT (Figure 3B,C). Accordingly, we measured the concentrations of SCFAs in the feces and serum samples of *Gnal* ^+/−^ and WT mice using targeted UHPLC‐MS/MS metabolite analysis. Compared to WT mice, significant reductions in butyric acid (BA) and isovaleric acid (IVA) levels were observed in fecal samples of *Gnal* ^+/−^ mice (**Figure** **5**A). In serum samples, the levels of vanillic acid (VA), 2‐methoxyvanillic acid (2MVA), butyric acid (BA), isobutyric acid (IBA), 2‐methylbutyric acid (2MBA), and hippuric acid (HPA) were significantly lower in *Gnal* ^+/−^ mice than WT mice (Figure 5B). Lower levels of BA were found in both feces and serum samples of *Gnal* ^+/−^ mice (Figure 5C). Furthermore, the BA levels in both feces and serum were found to be significantly correlated with spontaneous locomotor activity or motor coordination behaviors (Figure 5D,E). In parallel with the metabolomic profiles of *Gnal* ^+/−^ mice, after FMT, the BA were noticeably decreased in feces and serum of *Gnal* ^+/−^ recipient mice compared with WT recipient mice (Figure S3A,B, Supporting Information). Considering that butyrate is a well‐known SCFAs that could enhance the gut barrier integrity and stimulate *MUC2* mRNA expression,^[^ ^20^–^22^ ^]^ we then assessed *MUC2* expression in murine colon tissue using RT‐qPCR and found that *MUC2* mRNA expression was significantly decreased in the *Gnal* ^+/−^ recipient mice (Figure S3C, Supporting Information). Collectively, these results from microbiome, metabolomics, and FMT experiments support a close association of butyrate deficiency with the motor deficits in *Gnal* ^+/−^ mouse model of DYT25 dystonia.

### 2.5 Butyrate Supplementation Reverses Motor Deficits in Gnal+/− Mouse Model of DYT25 Dystonia

**Figure 6:**
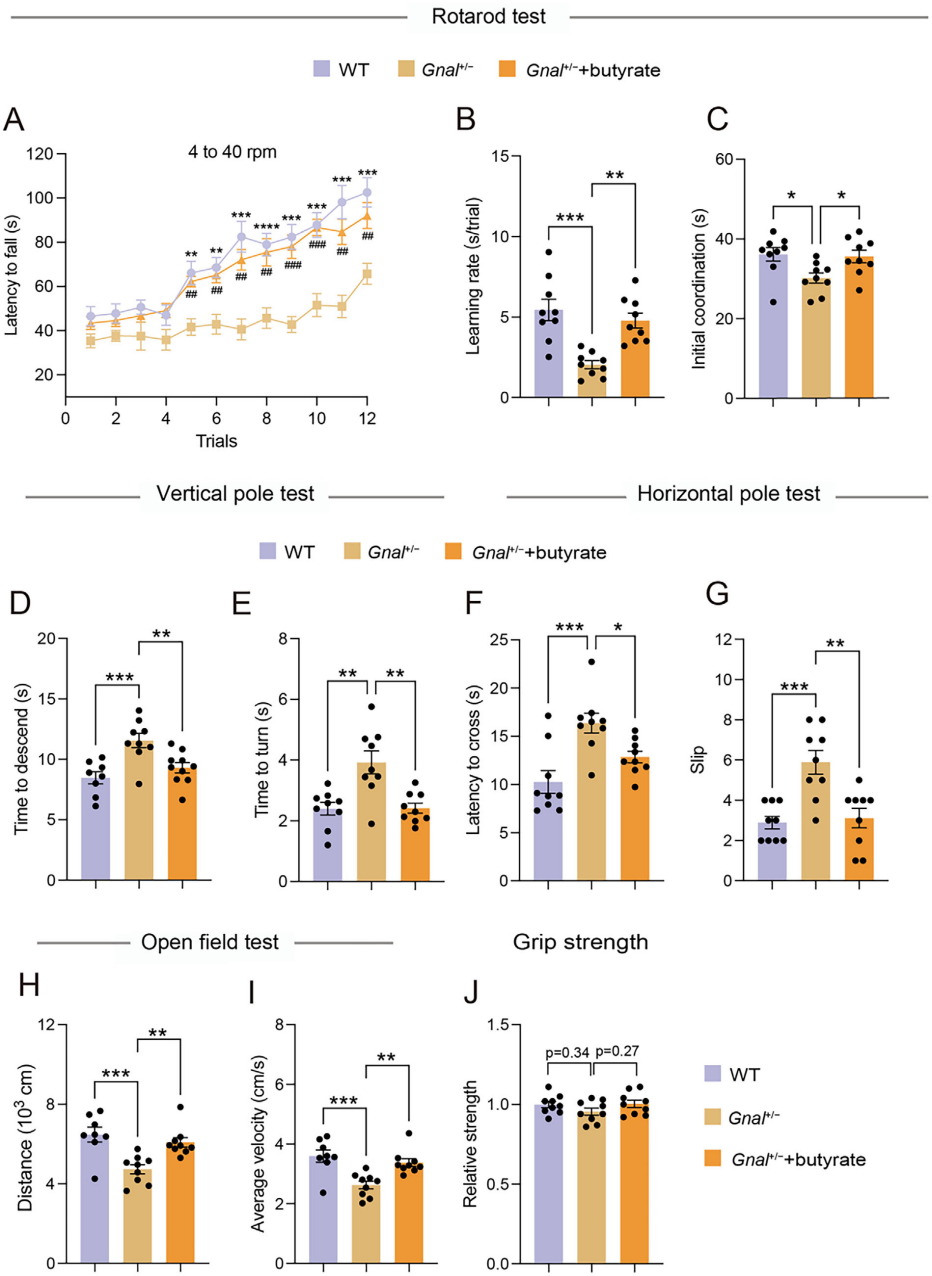
Butyrate supplementation reverses motor deficits in Gnal+/− mouse model of DYT25 dystonia. A) Rotarod performance (latency to fall) across 12 testing sessions of mice. B) Learning rate was derived from the slope value of linear regression performed on data from each individual mouse. C) Initial coordination derived from the intercept value of linear regression performed on data from each individual mouse. D,E) Time to descend (D) and time to turn (E) in the vertical pole test of mice. F,G) Latency to cross (F) and slip (G) in the horizontal pole test of mice. H,I) Spontaneous locomotor behavior of mice as assessed by the total distance (H) and average velocity (I). J) Grip strength of the four limbs of mice. All mice were 15–18 months of age. Data are shown as means ± SEM (n = 9 per group for A‐J). ^*^ *p* < 0.05, ^**^ *p* < 0.01, ^***^ *p* < 0.001, ^****^ *p* < 0.0001.

Next, we hypothesized that decreased levels of butyrate in the serum and feces of *Gnal* ^+/−^ mice could have an effect on behavioral outcomes. To answer this question, *Gnal* ^+/−^ mice were employed and orally administered with sodium butyrate or vehicle and examined for behavioral differences. Notably, butyrate treatment effectively rescued most of the symptoms of motor deficits in *Gnal* ^+/−^ mice. In the acceleration rotarod test, oral treatment with butyrate significantly increased the latency to fall in the overall trials and learning rate, as compared to the vehicle treatment group (**Figure** **6**A–C). Also, butyrate‐treated mice exhibited improved motor coordination in the two types of pole tests, as evidenced by decreased time to descend, time to turn, latency to cross, and slip (Figure 6D–G). Moreover, treatment with butyrate significantly improved the hypoactive spontaneous behavior, as indicated by increased total distance traveled and average velocity in *Gnal* ^+/−^ mice (Figure 6H,I). Meanwhile, there was no significant difference in muscle strength, body weight, and several serum parameters between butyrate‐treated and vehicle‐treated *Gnal* ^+/−^ mice (Figure 6J and Figure S4A–G, Supporting Information). Together, these results suggest that butyrate supplementation improved the motor deficits of *Gnal* ^+/−^ mice, supporting the hypothesis that decreased butyrate may contribute to manifestations of motor deficit behaviors in mice.

### 2.6 Butyrate Supplementation Modulates GABA Signaling and Increases the Striatal GABA Level

**Figure 7:**
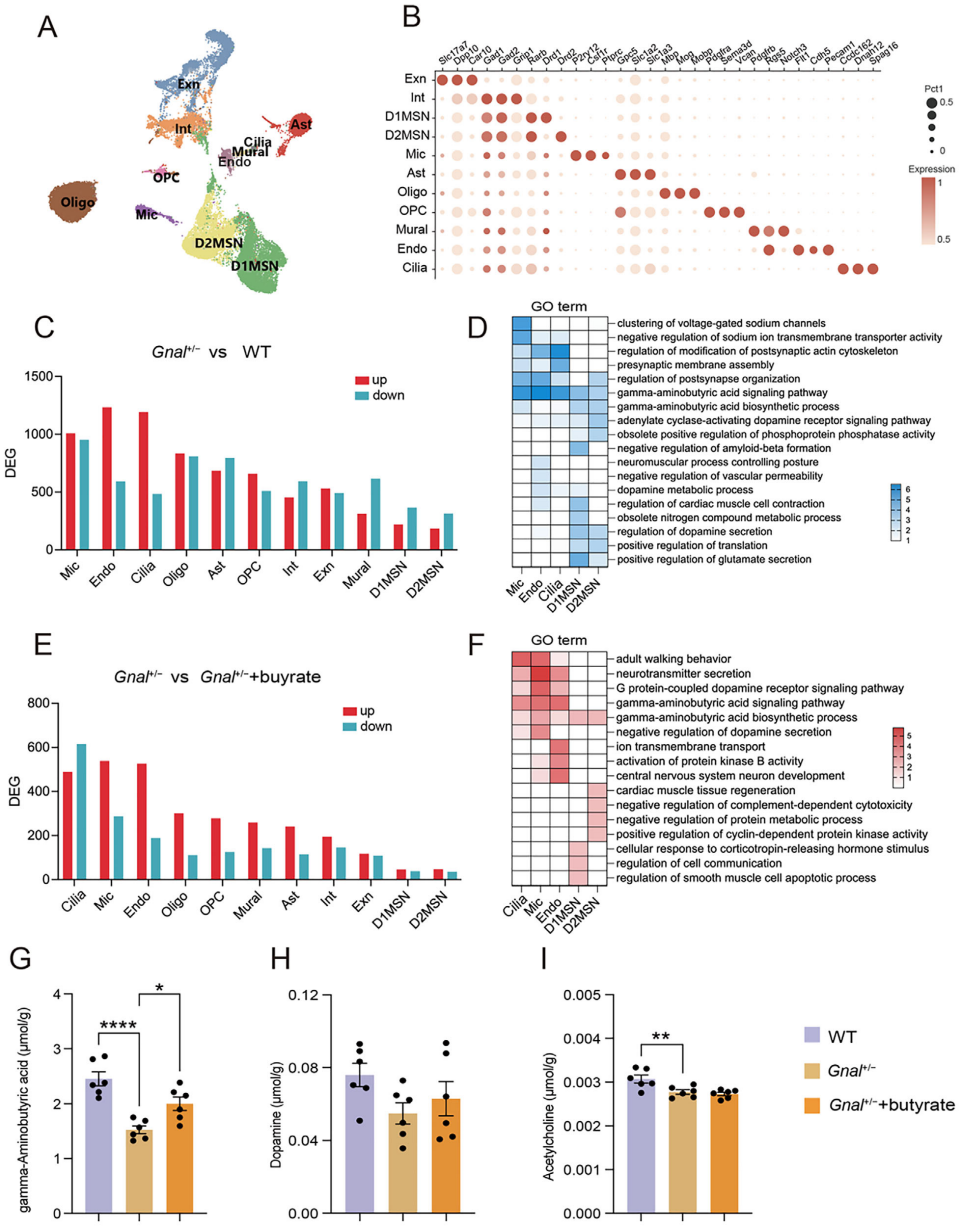
Butyrate supplementation modulates GABA signaling and increases the striatal GABA level. A) UMAP plot showing the major cell types in the striatum. B) Dot plot showing the expression of selective markers in different striatum cell types. C) Numbers of up‐ and downregulated DEG in each cell type between *Gnal* ^+/−^ and WT mice. D) Downregulated DEG between *Gnal* ^+/−^ and WT groups involved pathways in microglia, endothelial, ciliated, D1MSN, and D2MSN. E) Numbers of up‐ and downregulated DEG in each cell type between *Gnal* ^+/−^ and *Gnal* ^+/−^ +butyrate groups. F) Upregulated DEG between *Gnal* ^+/−^ and *Gnal* ^+/−^+butyrate groups involved pathways in microglia, endothelial, ciliated, D1MSN, and D2MSN. G–I) The levels of striatal GABA (E), acetylcholine (F), and dopamine (G) were measured by targeted metabolomics (n = 6 per group). Data are shown as means ± SEM. ^*^ *p* < 0.05, ^**^ *p* < 0.01, ^****^ *p* < 0.0001. UMAP, uniform manifold approximation and projection; Exn, excitatory neuron; Int, interneuron; Oligo, oligodendrocyte; Ast, astrocyte; Mic, microglia; OPC, oligodendrocyte precursors; Cilia, ciliated; Endo, endothelial.

We next sought to decipher the mechanisms by which the butyrate ameliorated the motor deficits of dystonia. Considering the fact that the striatum is tightly associated with dystonia, and that *Gnal* is particularly enriched in the striatum, we performed comparative transcriptome analyses of the striatum. Striatum samples from the three groups (WT, *Gnal* ^+/−^, and *Gnal* ^+/−^+butyrate) were isolated for nuclear extraction and snRNA‐seq. After raw data quality control (Figure S5A–E, Supporting Information), the cells for individual samples were shown (Figure S6A, Supporting Information). In the clustering analysis, we integrated cells from groups using unsupervised graph‐based clustering, which revealed 24 transcriptionally distinct cell clusters (Figure S6B, Supporting Information). Based on known marker gene expression, these clusters were annotated as D1MSN, D2MSN, and other cell types (**Figures** **7**A,B and S6C, Supporting Information), covering all of the major cell types known to be present in the striatum. By calculating the proportion of each cell type across groups, we found that *Gnal* ^+/−^ mice exhibited decreased proportions of D1MSN and D2MSN as well as increased proportions of oligodendrocytes and excitatory neurons, which were reversed by butyrate administration (Figure S6D, Supporting Information).

Comparison of the cellular transcriptional profiles between WT and *Gnal* ^+/−^ mice revealed that *Gnal* ^+/−^ mice displayed broad but distinct alterations in different cell types, with microglia, endotheliocytes, and ciliated cells exhibiting the highest number of differentially expressed genes (DEGs) (Figure 7C), suggesting that they may be the most affected cell types. In order to obtain insight into the functional consequence of transcriptional changes in the striatum, we conducted Gene Ontology (GO) analysis of DEGs from different cell types, including microglia, endotheliocyte, and ciliated cells, along with D1MSN and D2MSN, two cell types tightly associated with dystonia pathophysiology.^[^ ^23^, ^24^ ^]^ The GO analysis revealed that the terms related to the gamma‐aminobutyric acid (GABA) signaling pathway were enriched in multiple cell types (Figure 7D). Notably, transcriptome comparative analysis between cells from *Gnal* ^+/−^ and *Gnal* ^+/−^+butyrate groups revealed ciliated, microglia, and endotheliocyte cells exhibiting the highest number of DEGs (Figure 7E). The GO analysis further indicated that pathways involving GABA signaling, such as the gamma‐aminobutyric acid signaling pathway and gamma‐aminobutyric acid biosynthetic process, were enriched in *Gnal* ^+/−^ mice treated with butyrate (Figure 7F). For example, we found that DEGs in ciliated, microglia, endotheliocyte, D1MSN, and D2MSN were enriched in terms of gamma‐aminobutyric acid biosynthetic process, indicating that divergent transcriptional changes in different cell types may converge on the common pathway. Moreover, the RT‐qPCR analysis further confirmed marked upregulation of mRNA level of genes (*Gad1*, *Gad2, Vgat, Gabra2*, *Gabra4*, and *Gabrg3*) involved in GABA synthesis and transporter in striatum of *Gnal* ^+/−^ mice treated with butyrate (Figure S6E, Supporting Information).

Given the observed transcriptional changes (Figure 7D,F), we specifically measured striatal GABA levels using targeted metabolomics. Also, previous studies have suggested that dopamine and acetylcholine are closely related to dystonia,^[^ ^25^ ^]^ which led us to measure levels of these three neurotransmitters. Compared to the *Gnal* ^+/−^ mice, dietary supplementation with butyrate significantly restored striatal GABA levels, but not acetylcholine and dopamine levels (Figure 7G–I). Collectively, we propose that butyrate administration may modulate complex behaviors in *Gnal* ^+/−^ mice, with mechanisms that include regulation of GABA signaling and GABA levels in the striatum.

## 3 Discussion

Despite the considerable efforts spent in exploring the pathological mechanisms of dystonia, the whole picture of the pathophysiology of dystonia is still largely unknown, and there are limited insights into the role of gut microbiota in dystonia. In this study, we employed the *Gnal* ^+/−^ mouse model as it closely mimics the genetic status of patients with DYT25 dystonia and displays dystonia‐related behavior changes.^[^ ^11^, ^26^ ^]^ We found that the gut microbiota dysbiosis is sufficient to induce motor deficits of dystonia through the FMT experiment, raising the possibility that the gut microbiota is one of the targets of dystonia pathophysiology. Importantly, the *Gnal* ^+/−^ mice exhibited impaired butyrate metabolism and decreased butyrate levels in feces and serum, linking disrupted butanoate metabolism to dystonia susceptibility. The administration of butyrate alleviated the motor deficits of dystonia in *Gnal* ^+/−^ mice, concurrently induced favorable changes in GABA signaling, and increased the GABA levels in the striatum. These findings underline the contribution of gut dysbiosis in DYT25 dystonia and suggest a promising new strategy for treating dystonia through butyrate supplementation.

Dystonia is among the most prevalent movement disorders, though its prevalence estimates vary substantially across studies due to methodological heterogeneity and disparities in clinical recognition.^[^ ^27^, ^28^, ^29^ ^]^ Recent advancements in the study of other neurological disorders, such as PD, have shed light on the potential role of the gut‐brain connections.^[^ ^4^ ^]^ Recent findings have revealed a possible connection between the gut microbiota and dystonia. Ma et al reported that patients with isolated dystonia have significant gut dysbiosis, which might be related to altered serum metabolites.^[^ ^7^ ^]^ Timmners et al. surveyed the gut bacteria in patients of three subtypes of dystonia (cervical, dopa‐responsive, and 24 myoclonus dystonia) and observed differences compared to healthy controls.^[^ ^6^ ^]^ Additionally, a case report of a young woman with myoclonic dystonia showed a remarkable 90% improvement following antibiotic treatment.^[^ ^9^ ^]^ While these association studies in humans hint that gut bacteria might affect behavioral symptoms, a direct impact of the microbiota on the pathophysiology and behavioral outcomes of dystonia has not been described previously. We reported herein that colonization of antibiotic‐treated WT mice with gut microbiota from *Gnal* ^+/−^ mice, but not from WT controls, was sufficient to recapitulate motor deficits in dystonia (Figure 4A‐J). These discoveries confirm a direct link between gut dysbiosis and motor deficits of *Gnal* ^+/−^ mice.

The present study demonstrated that the gut microbiota of *Gnal* ^+/−^ mice was dominated by two phyla, namely Firmicutes and Bacteroidota (Figure 2H). This is in line with the previous studies by both Ma *et al.* ^[^ ^7^ ^]^ and Timmners *et al.*,^[^ ^6^ ^]^ which showed Firmicutes and Bacteroidota were the dominant phyla in dystonia patients. Specifically, Ma *et al.* ^[^ ^7^ ^]^ reported a decreased abundance of Bacteroidetes in dystonia patients compared to the healthy controls. Timmners *et al.* ^[^ ^6^ ^]^ did not observe any differences at the phylum level between dystonia patients and healthy controls. In the present study, there is no statistically significant difference in abundance of Bacteroidota or Firmicutes at the phylum level between *Gnal* ^+/−^and WT mice. These results suggest that the *Gnal* ^+/−^ mouse model may partially mirror the gut microbial profiles observed in the human dystonia condition.

Interestingly, we observed a large decrease in some butyrate‐producing bacteria in *Gnal* ^+/−^ mice, including the *Lachnospiraceae_NK4A136_group*, *Blautia*, and *Butyricicoccus*. Consistent with the alteration in butyrate‐producing bacteria, a marked decrease in the fecal and serum butyrate was observed in the *Gnal* ^+/−^ mice. *Lachnospiraceae_NK4A136_group*, representing a kind of butyrate‐producing bacteria, is a potential beneficial bacterium and is one of the main genera present in the intestine of mice. Members of the Lachnospiraceae family are considered the main producers of intestinal SCFAs, including butyrate.^[^ ^30^ ^]^ Recently, Lin *et al.* reported that 40.80% of the Lachnospiraceae genomes harbored complete butyrate pathways.^[^ ^30^ ^]^ The *Lachnospiraceae NK4A136 group* and its metabolite butyrate have been implicated in various biological processes. Wang *et al.* found that the upregulation of the levels of *Lachnospiraceae NK4A136* and butyrate may constitute an underlying mechanism responsible for the neuroprotective effects of melatonin in cognitive impairment caused by sleep deprivation.^[^ ^31^ ^]^ Similarly, Huang *et al.* identified *Lachnospiraceae_NK4A136_group* as a key genus enriched under high fermentable dietary fiber treatment and demonstrated that *Lachnospiraceae*‐derived butyrate could alleviate gut barrier impairment and reduce placental inflammation.^[^ ^21^ ^]^ In addition, Liu *et.al* reported that the abundance of *Blautia*, a genus of the Lachnospiraceae family, correlated with the clinical severity of PD. *B. producta* ATCC 27340 has the genes of butyrate kinase, which can convert butyryl‐CoA to butyrate and provide an alternative pathway to produce butyrate. Supplementation with butyrate‐producing bacterium *B. producta* ATCC 27340 improved motor dysfunction and neuroinflammation in a mouse model of PD.^[^ ^32^ ^]^ Some species of the *Butyricicoccus* genus, such as *B*. *pullicaecorum* and *B*. *porcorum*, are also well‐known butyrate‐producing bacteria and have been implicated in multiple diseases.^[^ ^33^, ^34^ ^]^

Currently, the development of new therapeutics is a top priority for dystonia research. Emerging data have shown that gut microbiota has advantages to modulate behaviors by producing neuroactive small molecules, such as SCFA.^[^ ^35^, ^36^ ^]^ In the present study, the gut microbiota of *Gnal* ^+/−^ mice exhibited a significant reduction in butyrate‐producing genera, such as *Lachnospiraceae_NK4A136*, *Blautia*, and *Butyricicoccus* (Figure 2I‐J), and the low abundance of these genera is reported to be associated with motor disorders such as PD.^[^ ^37^, ^38^ ^]^ Further microbial functions and metabolites analysis converged on disrupted butanoate metabolism, as evidenced by a significantly downregulated butanoate metabolism pathway and reduced butyrate levels in feces and serum of *Gnal* ^+/−^ mice (Figure 3, 5). Butyrate is considered as a health‐beneficial nutrient and is essential for maintaining the homeostasis of most cellular and physiologic processes.^[^ ^35^, ^39^ ^]^ Impaired butyrate production has been observed in both animal models and patients across a range of neurodegenerative diseases.^[^ ^19^, ^40^, ^41^ ^]^ Direct butyrate supplementation has been investigated as a therapeutic strategy for neurodegenerative conditions, including PD, HD, and spinocerebellar.^[^ ^42^, ^43^, ^44^ ^]^ In the present study, it was shown that butyrate supplementation beneficially improved motor deficits in *Gnal* ^+/−^ mice (Figure 6A‐J). These results provide a compelling experimental basis that butyrate can act as a mediator of gut‐brain communication in dystonia mouse models. As such, butyrate emerges as a promising candidate for microbiota‐centric anti‐dystonia therapies, warranting further investigation in future studies. Despite this promise, its clinical translation still faces many challenges owing to poor oral bioavailability, and foul smell, and taste. Alternative routes of butyrate administration, such as intrarectal delivery or continuous intravenous infusion, are often deemed unfeasible for patients with chronic disorders.^[^ ^45^, ^46^ ^]^ Innovative strategies, including nano‐carrier engineering and prodrugs, are expected to improve its systemic bioavailability and tissue specificity.^[^ ^47^, ^48^ ^]^ In the future, it is necessary to verify the results of animal studies in human trials and to explore the optimal dose and route of administration of butyrate.

Deciphering the neuroactive signals and pathways underlying the beneficial effect of the gut microbiota metabolites is vital. Considering the fact that a disturbance in the striatal transduction pathway may play a pivotal role in the pathogenesis of dystonia,^[^ ^49^ ^]^ we focus on transcriptional changes in the striatum to search for the molecular mechanism underlying butyrate‐mediated alleviation of dystonia. Using striatal snRNA‐seq, we found that compared to the vehicle‐treated *Gnal* ^+/−^ mice, GABA‐related signaling pathways were significantly upregulated across cell types in butyrate‐treated *Gnal* ^+/−^ mice (Figure 7F). In line with this, an elevated GABA level was observed in the striatum of butyrate‐treated mice (Figure 7G). RT‐qPCR analysis further demonstrated a dramatic restoration of critical signaling molecules in the GABA‐related signaling pathways, particularly including *Gad1*, *Gad2, Vgat, Gabra2*, *Gabra4*, and *Gabrg3* in the striatum of *Gnal* ^+/−^ mice treated with butyrate (Figure S6E, Supporting Information). These results suggested that the beneficial effects of butyrate on motor deficits in *Gnal* ^+/−^ mice may be associated with upregulated GABA signaling and elevated GABA levels in the striatum. GABA is a predominant neurotransmitter in the central nervous system, and preclinical and clinical evidence suggest that GABA signaling dysfunction might play a role in dystonia. Studies in animal models of primary dystonia, using pharmacologic, neurochemical, and autoradiographic measurements, reported alterations in GABA levels.^[^ ^50^, ^51^ ^]^ Enhancing the GABA level has been reported to mitigate dystonia. Hamann *et al.* demonstrated that in *dt^sz^* mutant Syrian golden hamsters, a model of paroxysmal dystonia, intrastriatal injections of GABA delayed the onset of dystonic attacks.^[^ ^52^ ^]^ Pharmacological and pathophysiological data in clinical evidence suggest that GABA signaling dysfunction is involved in different types of dystonia.^[^ ^53^, ^54^ ^]^ Patients with focal dystonia have reduced GABA levels in specific brain regions, particularly in the lentiform nucleus of the striatum, sensorimotor cortex, and hypothalamus, compared with healthy controls.^[^ ^55^, ^56^ ^]^ These findings in dystonia patients and animal models may suggest that reduced GABA signaling contributes to abnormal motor output of the disorder, while restoring GABA levels appears to be a promising therapeutic avenue for managing dystonia.^[^ ^25^, ^55^ ^]^

Recently, several lines of evidence strongly demonstrated a potential role of butyrate in the modulation of GABA. Sodium butyrate supplementation could increase GABA levels in the hypothalamus of PCOS rats and in the plasma of nonalcoholic fatty liver disease mice models, respectively.^[^ ^57^, ^58^ ^]^ Kratsman *et al* demonstrated that sodium butyrate induced an increase in the expression of genes involved in the GABA signaling pathway in the prefrontal cortex of BTBR mice model of autism and that a GABA reverse agonist decreased social behaviors in sodium‐butyrate‐treated BTBR mice, suggesting that sodium butyrate‐induced social behavior is specifically associated with changes in the GABA signaling pathways.^[^ ^59^ ^]^ It is well known that butyrate regulates various cell functions and exerts effects through distinct mechanisms, such as modulation of receptor signaling pathways or epigenetic regulation.^[^ ^31^, ^60^, ^61^ ^]^ Identifying the mechanism underlying the butyrate‐mediated GABA signaling in the striatum of *Gnal* ^+/−^ mice is of critical importance for achieving effective intervention, which indicates our future direction of further research.

This study has several limitations regarding mechanism investigation and clinical translation. Our studies were conducted in mice, and whether butyrate could also influence motor deficits of dystonia in humans remains to be determined. Though we have revealed the potential role of gut microbiota in the pathogenesis of DYT25 dystonia, future research to identify key microbial strains responsible for the observed metabolite changes will be essential. While our data from snRNA‐seq, targeted metabolomics, and RT‐qPCR point to a butyrate‐GABA signaling link, the direct electrophysiological evidence to demonstrate butyrate's modulation of GABAergic neurons is lacking. Consequently, the precise mechanism by which butyrate regulates GABA signaling in the striatal cell types remains to be elucidated. Apart from butyrate, which has been fully investigated in the present study, further exploration is needed to gain insight into other mechanisms linkages between gut microbiota and behavioral symptoms. Accordingly, future studies to address these key issues may help gain a better understanding of the pathogenesis of dystonia and explore the broader potential of butyrate in clinical treatment.

In conclusion, the present study provides compelling evidence for a modulatory role of the gut microbiota in the pathology of DYT25 dystonia, and butyrate supplementation alleviates the motor deficits of dystonia in *Gnal* ^+/−^ mice.

## 4 Experimental Section

### Animal

All animal procedures were approved by the Laboratory Animals Welfare and Animal Experimental Ethical Committee of the Institute of Zoology, Chinese Academy of Sciences (Approved No. IOZ‐IACUC‐2023‐286; Date: 25 August 2023). C57BL/6J mice (SPF, male, 6 weeks old) were purchased from Vital River Laboratory Animal Co., Ltd (Beijing, China). *Gnal* ^+/−^ mice (SPF, C57BL/6J background) were generated from GemPharmatech Co., Ltd (Nanjing, China). The *Gnal* ^+/−^ and *Gnal* ^+/+^ littermates were obtained from heterozygous (*Gnal* ^+/−^) breeding, as recommended for microbiome studies, ruling out potential differences in maternal care and/or early life effects on the gut microbiome.^[^ ^62^ ^]^ All the experimental mice were housed on a 12 h light/12 h dark cycle. For consistency, mice were fed the same normal chow (catlog no. 1025) provided by HFK Bioscience (Beijing, China).

### Behavioral Testing

On each experimental day, mice were taken to the behavioral test room and allowed to habituate for at least 1 h. Behavioral experiments were performed between 2:00. and 7:00 p.m. Following each test, the equipment was cleaned with 70% ethanol to avoid bias resulting from olfactory cues.

### Behavioral Testing—Open‐Field Test

Mice were gently put in a plastic open field arena (40 × 40 × 40 cm) and allowed to explore freely for 30 min. The locomotion of mice was continually monitored by a ceiling‐mounted camera, and the total distance and the amount of time spent were calculated using ViewPoint videotracking software (Labmaze V3.0) (ZhongShi Science & Technology, Beijing, China).

### Behavioral Testing—Rotarod Test

Assessment of motor coordination was performed using an accelerating rotarod apparatus, as described previously with some modifications.^[^ ^63^ ^]^ First, the mice underwent a training regimen consisting of two sessions over two consecutive days. On the first day, mice were placed in the rotarod apparatus three times, rotating at a constant speed of 4 rpm for 1 min each. On the second day, mice were placed in the rotarod three times for 2 min each, with the first min rotating at 4 rpm and the second min rotating at 8 rpm. Following the training sessions, the mice were challenged with the rotarod set to accelerate from 4 to 40 rpm over 5 minutes. The latency of the mouse falling from the rotating rod was calculated. A total of three trials were performed per mouse per day for four consecutive days.

### Behavioral Testing—Vertical Pole Test

The mouse was placed at the top of a rough‐surfaced pole (1 cm diameter and 60 cm high) and released when all four paws gripped the pole. The bottom of the pole was fixed to a home cage with the bedding present but without littermates. Animals would naturally tilt downward when placed on the pole and climb down the length of the pole to reach their home cage. During the two‐day acclimatization period before testing, each mouse was allowed to attempt to descend the pole and practice three times. On the test day, the time to completely orient the body downward (time to turn) and to climb down (time to descend) the home cage with all four paws was recorded. Each testing session consisted of three trials.^[^ ^64^ ^]^

### Behavioral Testing—Horizontal Pole Test

The horizontal pole test in mice was performed as described previously with some modifications.^[^ ^65^ ^]^ Animals were tested on a circular beam with a diameter of 1.5 cm. The beam was positioned 40 cm above a padded floor and 90 cm away from an enclosed box (20 cm square) that the mouse could escape. During a two‐day acclimatization training session, mice were placed at the beginning of the beam and trained to traverse the beam to the enclosed box three times. On the test day, the time taken to traverse the beam (latency to cross) and the number of times the hind feet slipped off on the beam (slip) were recorded for each trial. Each testing session consisted of three trials.

### Behavioral Testing—Grip Strength Test

Forelimb grip strength was measured using a grip strength meter (ZhongShi Science & Technology Co., Ltd, Beijing, China).^[^ ^66^ ^]^ The mice were placed on the grip strength meter, and the tail was gently pulled parallel to the ground until the mice lost grip. The peak grip strength was recorded by the meter automatically. Three trials were performed for each mouse with a 3 min rest in between.

### Gut Microbiota Depletion and Fecal Microbiota Transplantation (FMT)

Conventional mice (6‐week‐old, male, C57BL/6J) were administered with an antibiotic cocktail (ABX) in drinking water for 7 days, which consisted of ampicillin (1 g L^−1^), metronidazole (1 g L^−1^), neomycin sulfate (1 g L^−1^), and vancomycin (0.5 g L^−1^). The antibiotic‐containing water was replaced every other day. Then, the antibiotic solution was removed and replaced with drinking water for 1 day (wash‐out) before the transplantation. To prepare the sample for gavage, the fecal samples from donor mice (WT or *Gnal* ^+/−^ mice) of each group were collected, pooled, and resuspended in sterile ice‐cold saline (1 g mL^−1^ of feces). The solution was vigorously vortexed for 5 min, followed by natural sedimentation for 5 min. Recipient mice pretreated with antibiotics as described above were randomly divided into two groups. Then these mice were treated with bacterial suspension from each donor group (WT or *Gnal* ^+/−^ mice). The single gavage volume was 0.2 mL once a day for the first 3 days, and 3 times weekly later. Behavioral experiments were initiated at 9 weeks post‐transplantation.

### Sodium Butyrate Treatment

*Gnal* ^+/−^ and *Gnal* ^+/+^ littermates mice, aged at 15–18 months, were randomly divided into groups: 1) *Gnal* ^+/+^ mice + saline, 2) *Gnal* ^+/−^ mice + saline, or 3) *Gnal* ^+/−^ mice + butyrate. The sodium butyrate was administered orally at a dose of 1000 mg kg^−1^ body weight once a day for 12 weeks, while the control group received an equivalent volume of saline, and the behavioral tests were performed on the last week.

### Bio‐Sample Collection

Following the behavioral tests, the mice were sacrificed by cervical dislocation under isoflurane anesthesia. Blood samples were collected and centrifuged to obtain serum. The colon and gastrocnemius tissues were carefully dissected and immediately frozen in liquid nitrogen. The whole brain was isolated, and the striatum was microdissected out on a cold plate. All samples were then stored at −80°C until further processing. For gut microbiome profiling, fresh fecal pellets were collected and snap frozen before analysis.

### Histopathological Analysis

The colon and gastrocnemius tissues were freshly collected and immersed in 4% paraformaldehyde (Servicebio, Cat# G1101, China) and fixed for 24 h. For hematoxylin & eosin (H&E) staining, the tissue was then embedded in paraffin and sliced into 5 µm sections, which were processed with conventional hematoxylin/eosin staining. For alcian blue staining, the tissue sections were treated with alcian blue dye solution, incubated with alcian blue solution for 10–15 min, and then treated with Nuclear Fast Red dye solution for 3 min. Slides were rinsed in running tap water and then dehydrated with an ascending ethanol gradient (70%, 95%, and 100%). For wheat germ agglutinin (WGA) staining, the section was incubated with WGA‐conjugated and 4, 6‐diamidino‐2‐phenylindole in the dark for 2 h. Microscopic examination was performed using an optical microscope (NIKON ECLIPSE E100, Nikon, Tokyo, Japan). Quantification was performed using ImageJ software (NIH, USA).

### Immunohistochemical Staining

For immunohistochemical staining for claudin‐1, muc2, occludin‐1, and zo‐1, EDTA (pH 9.0) was prepared for antigen retrieval, and the sections were boiled for 9 min, broken for 8 min, and medium heat for 7 min. Incubation of the sections in 3% H_2_O_2_ for 25 min inhibited endogenous peroxidase activity in the section. Non‐specific sites were blocked in 10% goat serum. Each slide was incubated with anti‐mouse (Servicebio, Cat# GB12032, China), anti‐mouse muc2 (Servicebio, Cat# GB11344, China), anti‐mouse occludin (Servicebio, Cat# GB111401, China), and anti‐mouse zo‐1 (Servicebio, Cat# GB111402, China) antibody overnight, followed by HRP‐conjugated goat anti‐mouse IgG (Servicebio, Cat# GB23301, China) incubation for 45min at room temperature. Diaminobenzidine tetrahydrochloride (Servicebio, Cat# G1212, China) was applied to visualize the immunoreactivity. The slides were dehydrated and sealed with neutral resin after hematoxylin staining. Slides were observed using a NIKON ECLIPSE E100 microscope (Nikon, Japan) and quantified using ImageJ software (NIH, USA).

### Targeted Metabolomics—SCFA

Feces or serum samples were extracted by 50% acetonitrile, and vortexed for 2 min, and centrifuged at 15000 × *g* for 15 min (4°C). Then, the supernatant and mixed with acetonitrile, 3‐nitrophenylhydrazine, EDC, and pyridine solution. React at 40°C for 30 min, add 1% formic acid to terminate the reaction at room temperature for 5 min. The centrifuge and the supernatant were transferred for LC‐MS analysis. Mix prepared samples for the quality control sample. The UHPLC‐MS/MS analysis was performed on an Agilent 1290 Infinity II UHPLC system coupled to a 6470A Triple Quadrupole mass spectrometry (Santa Clara, CA, United States). The mobile phase A was deionized water with 0.1% formic acid and the mobile phase B was acetonitrile with 0.1% formic acid. Samples were injected onto an Acquity UPLC BEH C18 column (100mm × 2.1mm, 1.7µm). The eluted analysts were ionized in an electrospray ionization source in negative mode (ESI‐). The mass spectrometric parameters are as follows: drying gas temperature (300°C), gas flow rate (5 L min^−1^), nebulizer pressure (45 psi), sheath gas temperature (300°C), and flow rate (11 L min^−1^). MassHunter software (version B.08.00, Agilent) was used to collect data.

### Targeted Metabolomics—Neurotransmitter

Striatum samples were extracted using 80% methanol at a volume of 10 times the sample mass (Vm, µL mg^−1^), then homogenized with beads at 40 Hz for 4 min using a Tissuelyser (JX‐24, Jingxin, Shanghai, China). The resulting homogenate was centrifuged at 15000 × *g* and 4°C for 15 min. 20 µL supernatant was applied to be derivatized with 50 µL sodium carbonate (100mM) and 30 µL benzoyl chloride (2% in acetonitrile) at room temperature for 10 min. After centrifugation, the supernatant was isometrically mixed with stable isotope‐labeled internal standards for the quantification of amino acids and neurotransmitters. The UHPLC‐MS/MS analysis was performed on an Agilent 1290 Infinity II UHPLC system coupled to a 6470A Triple Quadrupole mass spectrometry (Santa Clara, CA, United States). The mobile phase A was deionized water with 0.1% formic acid and 10 mm ammonium formate, and the mobile phase B was pure acetonitrile. Samples were injected onto an Acquity UPLC BEH C18 column (100mm × 2.1mm, 1.7µm). The eluted analysts were ionized using an electrospray ionization source in the positive mode (ESI+). The mass spectrometric parameters are as follows: drying gas temperature (300°C), gas flow rate (5 L min^−1^), nebulizer pressure (45 psi), sheath gas temperature (300°C), and flow rate (11 L min^−1^). MassHunter software (version B.08.00, Agilent) was used to collect data.

### rRNA Sequencing

Fresh fecal samples were collected in the morning (8:00–9:00 a.m.) into 2 mL tubes, quickly snap‐frozen and stored in −80°C until DNA extraction. The microbial DNA was extracted from the fecal samples using a E.Z.N.A.® Soil DNA Kit (Omega Bio‐tek, USA) according to the manufacturer's instructions. The DNA quality and concentration were measured by agarose gel electrophoresis and NanoDrop2000 before downstream high‐throughput sequencing. For 16S rRNA sequencing, variable regions 3 and 4 (V3‐V4) of the 16S rRNA gene were amplified using 338F and 806R primers and sequenced on Illumina Miseq PE300 (Illumina, USA). The amplification was controlled for purity with a non‐template negative control for each sample. The Illumina MiSeq sequencing platform and PE300 chemicals (Majorbio Biomedical Technology Co, Shanghai, China) were used for paired‐end amplicon sequencing. The raw data were analyzed on the Majorbio Cloud platform (https://www.majorbio.com). A total of 1088,769 reads were generated, averaging 68,048 ± 4,826 (mean ± standard deviation) reads per sample. Quality filtering was executed using fastp (0.19.6) by employing truncating reads at the first base where the average quality score in a sliding 50‐bp window fell below 20. Following quality trimming, reads shorter than 50 bp were discarded. Additionally, any reads containing N were also removed. Then the resulting sequences were merged with FLASH (v1.2.11), and uploaded to QIIME2 (version 2022.2) for removing chimera sequences and amplicon sequence variant (ASV) evaluation using the DADA2 plugin. Taxonomic assignment of ASVs was performed using the Naive bayes consensus taxonomy classifier implemented in Qiime2 and the SILVA 16S rRNA database (v138). The alpha diversity indexes of ASVs were calculated using Mothur v1.30. Beta diversity analysis was performed using UniFrac distance metrics and visualized via principal coordinate analysis (PCoA). Differences in the UniFrac distances between groups were determined by permutational multivariate analysis of variance (PERMANOVA) using the Vegan package in R (version 3.3.1). Differential bacterial abundance analysis calculated by Welch's *t*‐test using two filters (FC > 1, *p* < 0.05). RandomForest analysis was constructed by the Vegan package in R (version 3.3.1) with contributing >2% to classification between WT and *Gnal* ^+/−^ mice. Phylogenetic Investigation of Communities by Reconstruction of Unobserved States (PICRUSt) based on ASVs was employed to predict the abundances of functional categories using Kyoto Encyclopedia of Genes and Genomes (KEGG) orthologs (KO).

### Single Nuclei RNA Sequencing (snRNA‐seq)

For snRNA‐seq, the striatum was dissected and quickly frozen in liquid nitrogen to retain intact nuclei. The nuclei were isolated and purified as previously described with modifications.^[^ ^67^ ^]^ The frozen tissues were homogenized in nuclei lysis buffer (NLB). The snRNA‐Seq libraries were generated using the 10X Genomics Chromium Controller Instrument and Chromium Single Cell 3’ V3.1 Reagent Kits (10X Genomics, Pleasanton, USA). DNA library quality was measured by Qubit High Sensitivity DNA assay (Thermo Fisher Scientific), and sequenced on an Illumina Novaseq6000 as described.^[^ ^68^ ^]^ The scRNA‐seq data analysis was performed by NovelBio Co., Ltd. with NovelBrain Cloud Analysis Platform. Briefly, raw reads were trimmed to remove low‐quality bases and adapter sequences using Fastp (v.0.18.0) 26. Feature‐barcode matrices were obtained by aligning reads to the mouse genome (mm10 Ensemble: version 100) using CellRanger v7.1.0. On average, more than 115G sequencing reads were obtained for each sample, with a median sequencing saturation of 56.30% (56.00–57.8%). The mean reads detected per cell was 31,278. Cells contained over 200 expressed genes, and the mitochondria UMI rate below 5% passed the cell quality filtering, and mitochondria genes were removed in the expression table. The Seurat package (version 4.1.1) was used for cell normalization based on the expression matrix according to the UMI counts of each sample and the percent of mitochondria rate to obtain the scaled data. PCA was constructed based on the scaled data with the top 2000 highly variable genes, and the top 10 principals were used for tSNE construction and UMAP construction. Utilizing a graph‐based cluster method, the unsupervised cell cluster result was acquired based on the PCA top 10 principal and the marker genes were calculated by FindAllMarkers significance was defined by the *P* value and FDR. Nuclei were classified into 24 clusters using UMAP (Uniform Manifold Approximation and Projection for Dimension Reduction), and the major cell types were further annotated using known genetic markers. The DEG was calculated by the FindMarkers function (Seurat), with the Wilcoxon rank‐sum test algorithm using the following criteria: 1) log2 FC > 0.25 or log2 FC < −0.25; 2) *P* value < 0.05; 3) min.pct > 0.1. Gene ontology analysis was conducted by retrieving and mapping gene sets using GO (geneontology.org), and Fisher's exact test was applied to identify the significant GO categories and a *P* value < 0.05 and an FDR of *q* value < 0.05 (with Holm‐Bonferroni correction) were used as cutoff values for significance.

### Quantitative Real‐Time PCR (RT‐qPCR)

RNA was extracted from the colon and striatum by TRIzol reagent (Blotopped, Beijing, China), and then an RNA reverse transcription procedure was performed (TIANGEN, Beijing, China). A Pangaea real‐time PCR system (Aperbio, Suzhou, China) was used to perform qPCR analysis, accompanied by TIANGEN SYBER Green Supermix (Beijing, China). The mRNA expression was normalized using β‐actin expression. The primer sequences are listed in Table S1 (Supporting Information).

### Statistical Analysis

All values are presented as mean ± standard error of the mean (SEM). An unpaired two‐tailed Student's *t*‐test was used for the comparison with one variable. Differences between multiple groups with one variable were determined using one‐way ANOVA followed by Tukey's post hoc test. To compare multiple groups with more than one variable, two‐way ANOVA followed by Tukey's or Bonferroni's post hoc test. All statistical analyses were conducted using GraphPad Prism 10 (GraphPad, USA), and the significance was set at ^*^ *p* < 0.05, ^**^ *p* < 0.01, ^***^ *p* < 0.001, and ^****^ *p* < 0.0001.

## Author Contributions

TT and KH designed and supervised the study. JG performed experiments and analyzed the data. JG and TT wrote the manuscript. TT contributed to text revision and discussion. TT, KH, and SK reviewed the manuscript. All authors have read and agreed to the published version of the manuscript.

## Conflict of Interest

The authors declare no competing interests.

## Supporting information



Supporting Information

Supplemental Table 1

## Data Availability

Supplementary information is available in Additional file 1. The raw sequence data of 16S rRNA gene sequencing and snRNA sequencing reported in this study have been deposited in the Genome Sequence Archive (Genomics, Proteomics & Bioinformatics 2021) in National Genomics Data Center (Nucleic Acids Res 2022), China National Center for Bioinformation / Beijing Institute of Genomics, Chinese Academy of Sciences (BioProject: PRJCA038939) that are publicly accessible at (https://ngdc.cncb.ac.cn/bioproject/browse/PRJCA038939).
