# Supplementary material for: The Gut Microbiota Regulates Motor Deficits via Butyrate in a Gnal +/− Mouse Model of DYT25 Dystonia
Source: Adv Sci (Weinh). 2025 Dec 12;13(11):e12942. doi: 10.1002/advs.202512942 (PMC12931183; doi:10.1002/advs.202512942)
Supplement: Supplementary file 1 — Supporting Information [file ADVS-13-e12942-s001.docx]

**Supplementary Data**

**Supplementary Table**

**Supplementary Table 1. Primer sequences used for RT-qPCR**

| **Target Genes** | **Forward Primer 5′–3′** | **Reverse Primer 5′–3′** |
| --- | --- | --- |
| *Gad1* | CTCAGGCTGTATGTCAGATGTTC | AAGCGAGTCACAGAGATTGGTC |
| *Gad2* | TCAACTAAGTCCCACCCTAAG | CCCTGTAGAGTCAATACCTGC |
| *Vgat* | GCCATTCAGGGCATGTTCG | TGAGGATCTTGCCGGTGTAG |
| *Gabra2* | GCTACGCTTACACAACCTCAGA | GACTGGCCCAGCAAATCATACT |
| *Gabra4* | AGAACTCAAAGGACGAGAAATTGT | TTCACTTCTGTAACAGGACCCC |
| *Gabrg3* | AATACATCCAGATTCCACAAGATG | CACAGGTGTCCTCAAATTCCT |
| *Muc2* | GGGAATGTTGCAAGAAGTGC | TTTTGTGAATCTCCCCAGGC |
| *β-actin* | GGCTGTATTCCCCTCCATCG | CCAGTTGGTAACAATGCCATGT |

**Supplementary Figures**

**
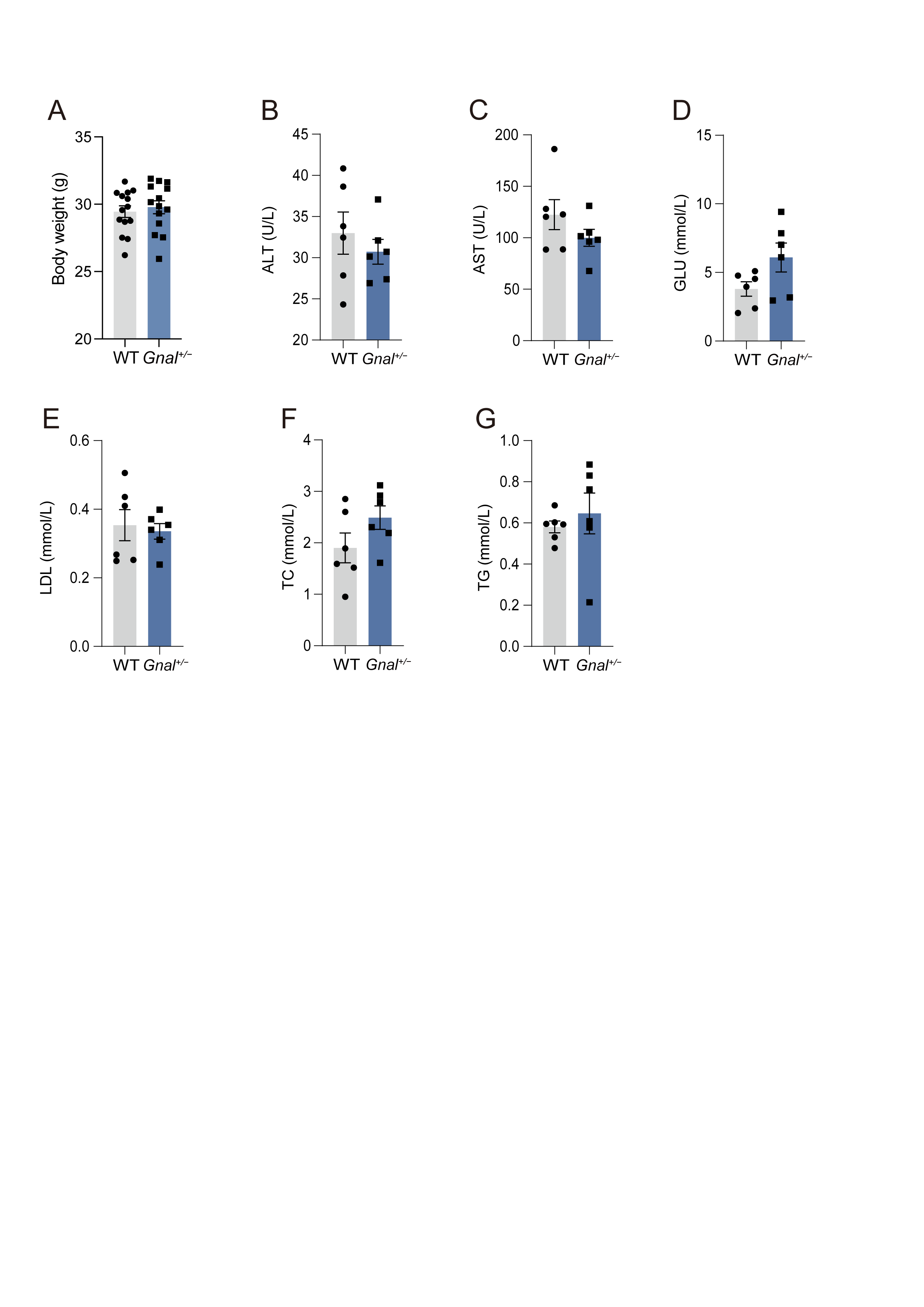
**

**Supplemental Figure 1****.** **The** **body weight and several serum chemistry parameters of** ***Gnal*^+/−^ mice,** **relative to Figure 1.**

(A) Body weight of *Gnal*^+/−^ and WT mice (n=14 per group). (B–G) Blood biochemical parameters include ALT (B), AST (C), GLU (D), LDL (E), TC (F), and TG (G) of mice (n=6 per group). ALT, alanine aminotransferase; AST, aspartate transaminase; GLU, glucose; LDL, low density lipoprotein; TC, total cholesterol; TG, triglycerides.

**
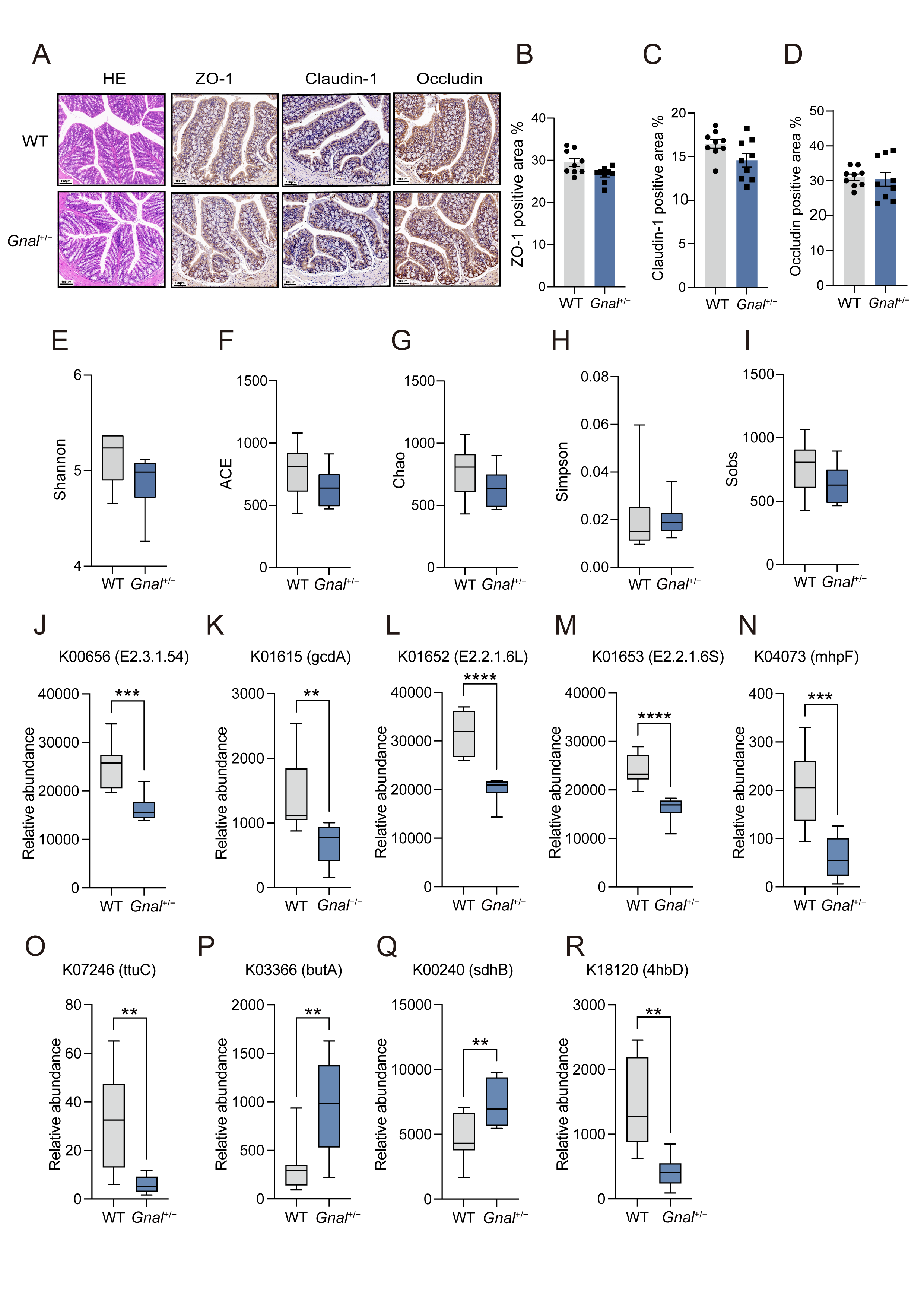
**

**Supplemental Figure 2.** **Colonic histological staining and gut microbial indexes** **of *Gnal*^+/−^ mice, relative to Figure 2-3.**

(A) Representative images of HE staining and immunohistochemistry staining for ZO-1, Claudin-1, and Occludin sections in colonic tissue sections (scale bars = 100 μm). (B–D) Quantification of ZO-1 (B), Claudin-1 (C), and Occludin (D) positive area in colonic sections (n=9 slices from 3 mice). (E–I) Box plots showing the α diversity of gut bacteria from WT and *Gnal*^+/−^ mice (n = 8 per group), which were measured by Shannon, ACE, Simpson, Chao, Simpson, and Sobs. (J-R) Box plots of relative abundance of KOs in the butyrate metabolism (n = 8 per group). ***p* < 0.01, ****p* < 0.001, *****p* < 0.0001. HE, hematoxylin-eosin; KO, KEGG orthology.

**
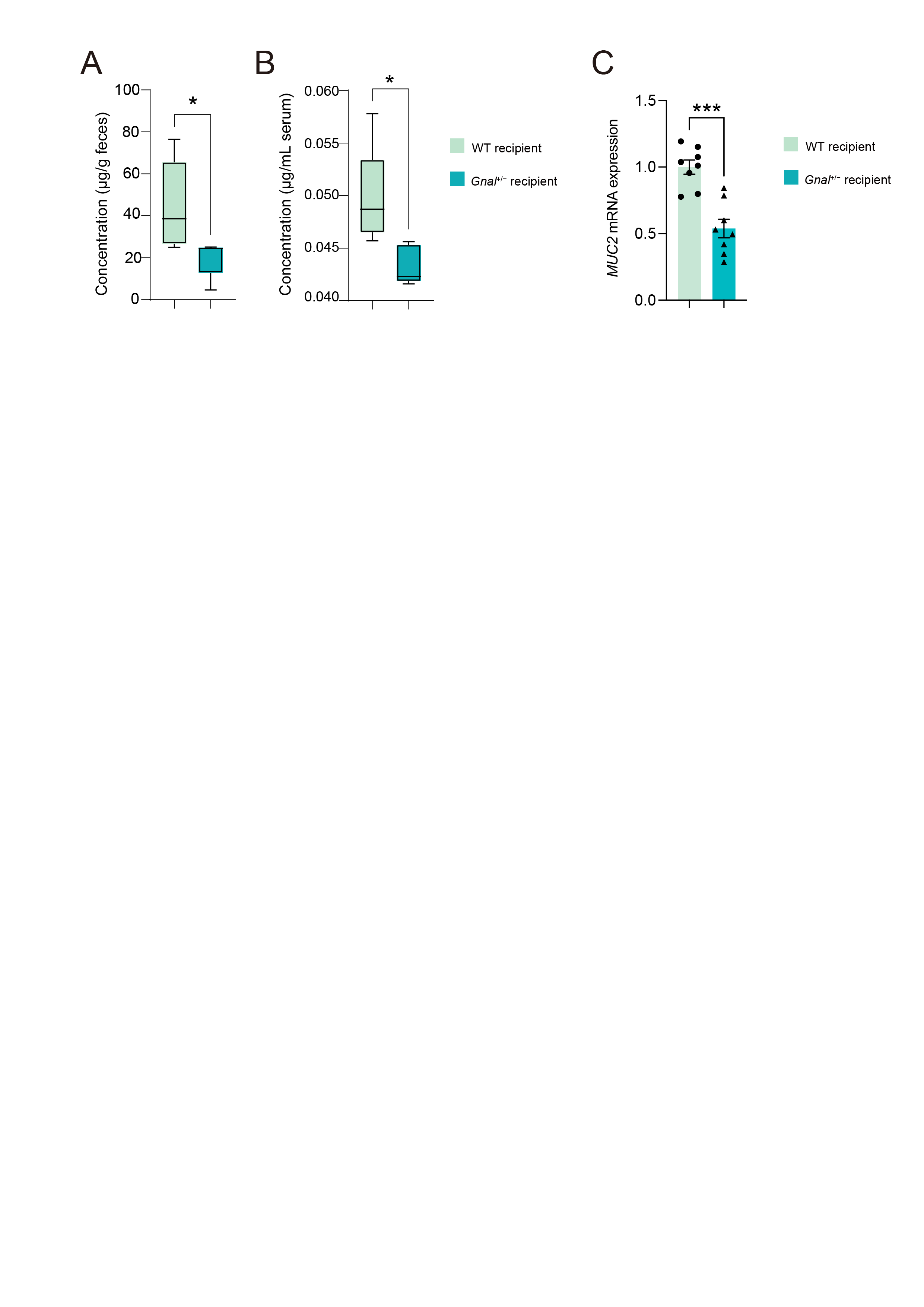
**

**Supplemental Figure 3 After FMT,** **butyrate levels and the *MUC2* mRNA level**

**were reduced in *Gnal*^+/−^ recipient mice, relative to Figure 5.**

(A, B) The concentration of BA in feces (A) and serum (B) samples of WT recipient and *Gnal*^+/−^ recipient mice (n=5 per group). (C) Relative *MUC2* mRNA expression level in the colon of mice (n=8 per group). **p* < 0.05, ****p* < 0.001. BA, butyric acid.

**
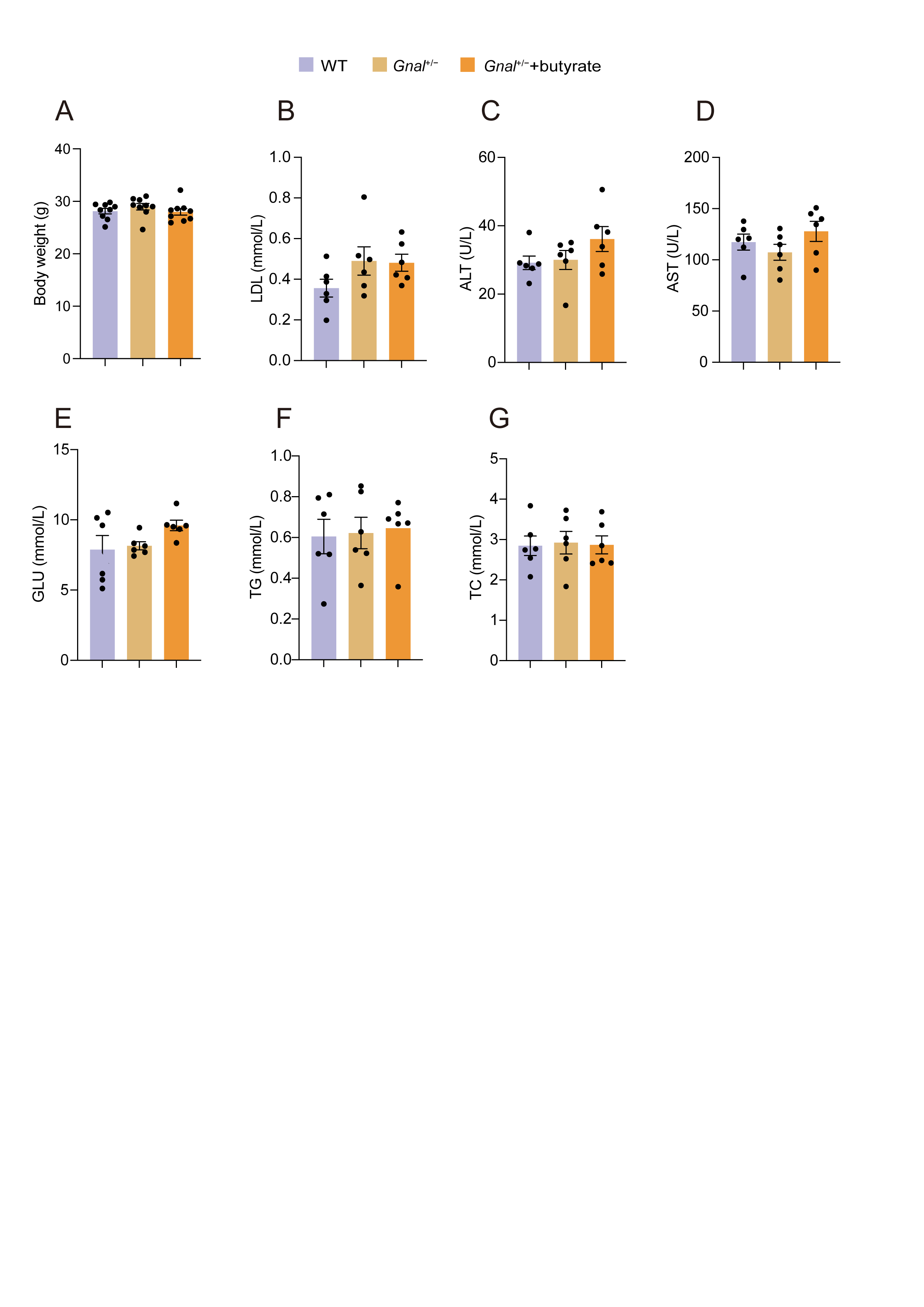
**

**Supplemental Figure 4 The body weight and several serum chemistry parameters of *Gnal*^+/−^ mice supplemented with butyrate,** **relative to Figure 6.**

(A) Body weight of mice (n=9 per group). (B–G) Blood biochemical parameters include LDL (B), ALT (C), AST (D), GLU (E), TG (F), and TC (G) (n =6 per group). ALT, alanine aminotransferase; AST, aspartate transaminase; GLU, glucose; LDL, low density lipoprotein; TC, total cholesterol; TG, triglycerides.

**
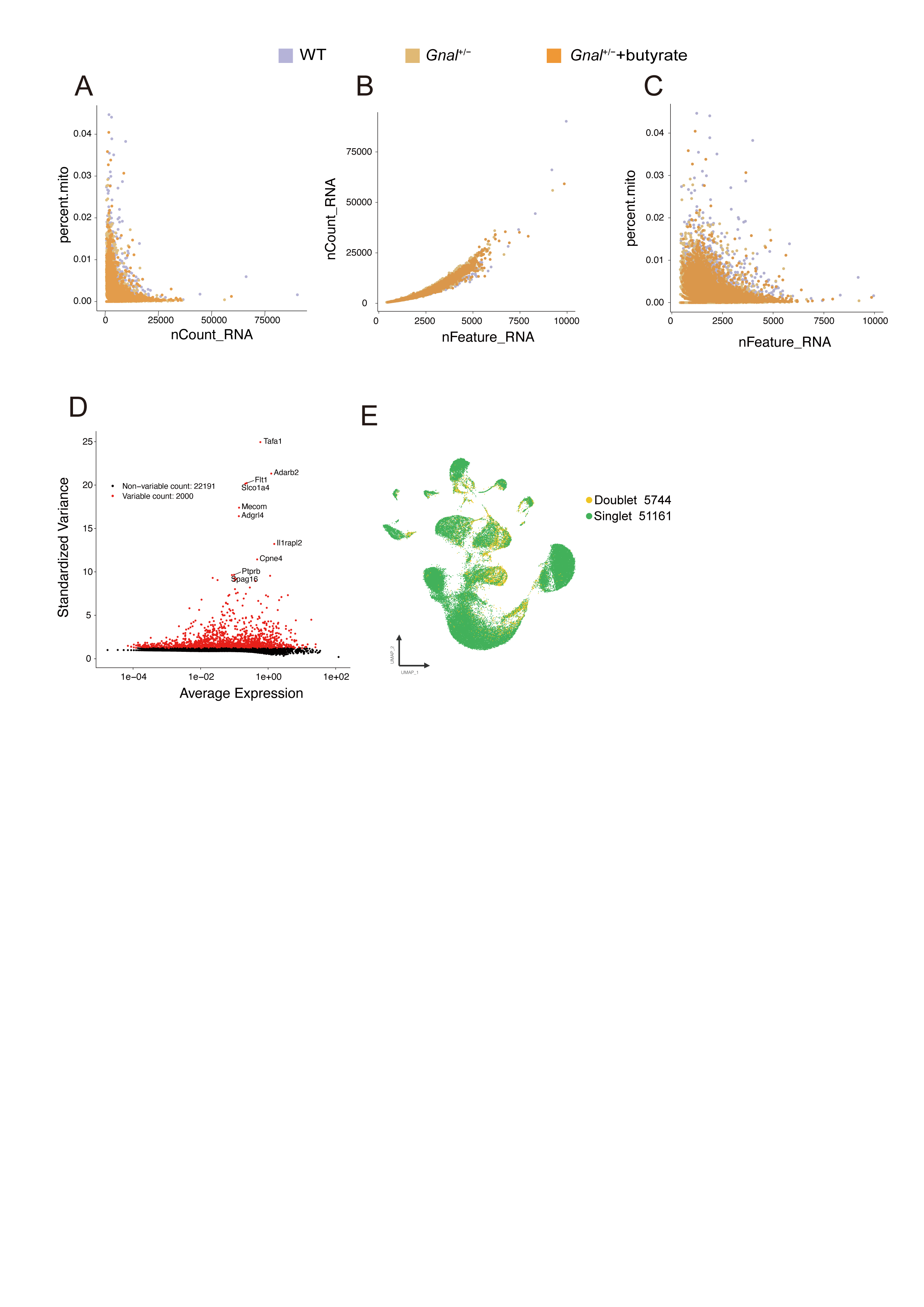
**

**Supplemental Figure 5 QC analysis of single-nucleus RNA sequencing****, relative to Figure 7.**

(A) Scatter plot comparing the mitochondria percentage (percent.mito). (B) Scatter plot comparing the number of RNA features (nFeature_RNA). (C) Scatter plot comparing absolute UMI counts (nCount_RNA). (D) top 2000 variable genes in total genes. (E) Doublets and single nuclei were identified.

**
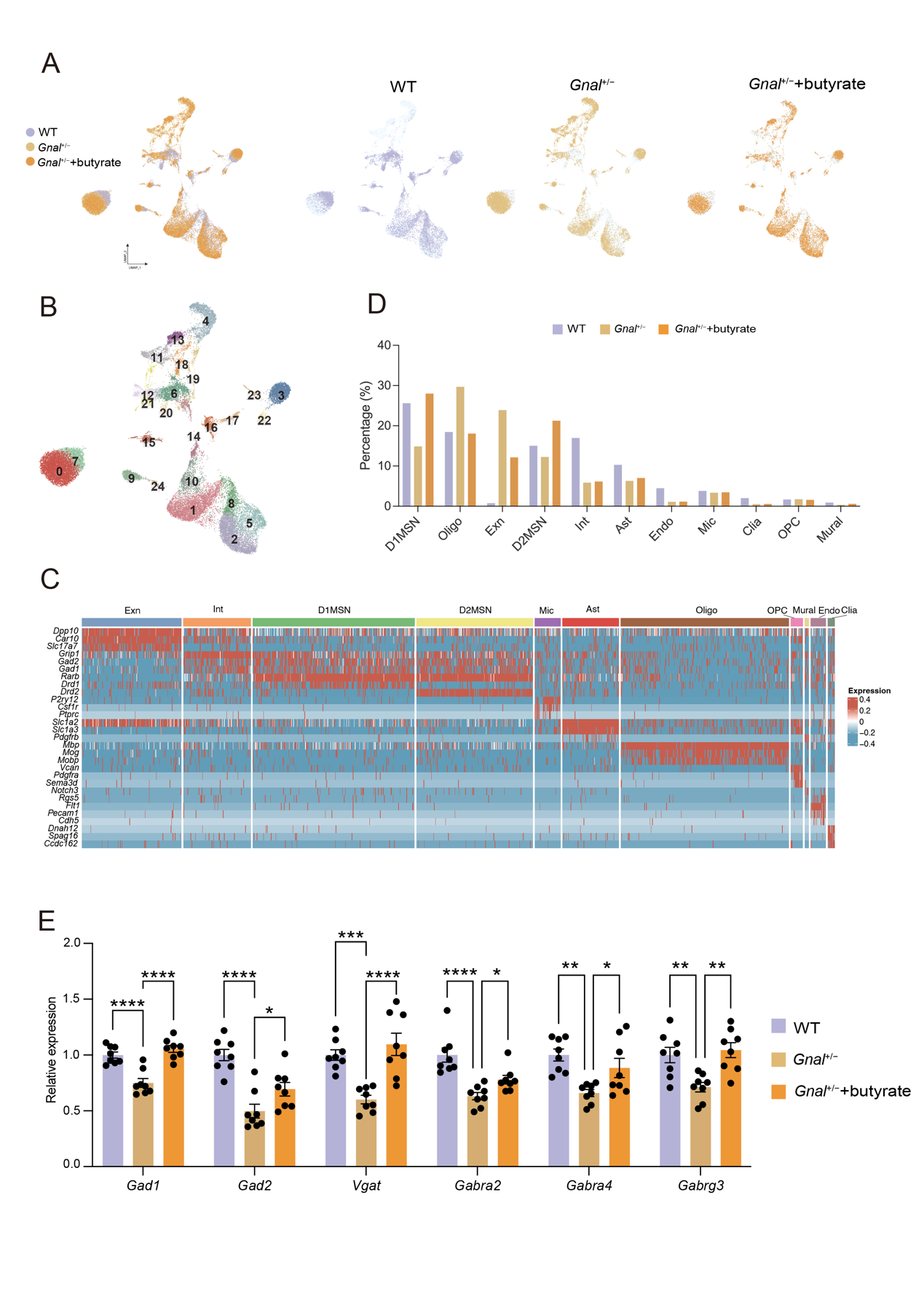
**

**Supplemental Figure 6 Striatal cell type-specific transcriptional changes and the relative expression level of genes involved in the GABA signaling pathway in the striatum of mice, relative to Figure 7.**

(A) Dissected UMAP plot showed a coincident distribution of nuclei clustering in the three groups. (B) UMAP visualization shows clustering of nuclei. (C) Heatmap showed the expression levels, distribution of representative cell-type markers, and the numbers annotated by cell types. (D) The proportion of the cell types in each group. (E) Relative expression level of genes involved in the GABA signaling pathway in the striatum of mice (n=8 per group). UMAP, uniform manifold approximation and projection; Exn, excitatory neuron; Int, interneuron; Oligo, oligodendrocyte; Ast, astrocyte; Mic, microglia; OPC, oligodendrocyte precursors; Cilia, ciliated; Endo, endothelial. *p < 0.05, ***p* < 0.01, ****p* < 0.001, *****p* < 0.0001.
