# Supplementary material for: The Gut Microbiota Regulates Motor Deficits via Butyrate in a Gnal +/− Mouse Model of DYT25 Dystonia
Source: Adv Sci (Weinh). 2025 Dec 12;13(11):e12942. doi: 10.1002/advs.202512942 (PMC12931183; doi:10.1002/advs.202512942)
Supplement: Supplementary file 2 — Supplemental Table 1 [file ADVS-13-e12942-s002.docx]

**Supplementary Data**

**Supplementary Table**

**Supplementary Table 1. Primer sequences used for RT-qPCR**

| **Target Genes** | **Forward Primer 5′–3′** | **Reverse Primer 5′–3′** |
| --- | --- | --- |
| *Gad1* | CTCAGGCTGTATGTCAGATGTTC | AAGCGAGTCACAGAGATTGGTC |
| *Gad2* | TCAACTAAGTCCCACCCTAAG | CCCTGTAGAGTCAATACCTGC |
| *Vgat* | GCCATTCAGGGCATGTTCG | TGAGGATCTTGCCGGTGTAG |
| *Gabra2* | GCTACGCTTACACAACCTCAGA | GACTGGCCCAGCAAATCATACT |
| *Gabra4* | AGAACTCAAAGGACGAGAAATTGT | TTCACTTCTGTAACAGGACCCC |
| *Gabrg3* | AATACATCCAGATTCCACAAGATG | CACAGGTGTCCTCAAATTCCT |
| *Muc2* | GGGAATGTTGCAAGAAGTGC | TTTTGTGAATCTCCCCAGGC |
| *β-actin* | GGCTGTATTCCCCTCCATCG | CCAGTTGGTAACAATGCCATGT |
